# Evidence of kinship, overwintering, and *Wolbachia* presence in *Aedes albopictus* in urban areas and points of entry in the Netherlands

**DOI:** 10.1186/s13071-026-07415-z

**Published:** 2026-05-13

**Authors:** Adolfo Ibáñez-Justicia, Federica Lucati, Paulina Maria Lesiczka, Tim Warbroek, Vicente J. Ontiveros, Jenny Caner, Rubén Bueno-Marí, Carles Aranda, Alessandro Albieri, Bart van de Vossenberg, John R. B. Palmer, Frederic Bartumeus, Marc Ventura, Arjan Stroo

**Affiliations:** 1 Centre for Monitoring of Vectors (CMV), Netherlands Institute for Vectors, Invasive Plants and Plant health (NIVIP), Netherlands Food and Consumer Product Safety Authority (NVWA), Wageningen, the Netherlands; 2 Centre d’Estudis Avançats de Blanes (CEAB-CSIC), Girona, Blanes, Spain; 3 Department of Political and Social Sciences, Universitat Pompeu Fabra (UPF), Barcelona, Spain; 4 National Plant Protection Organization (NPPO-NL), Netherlands Institute for Vectors, Invasive Plants and Plant health (NIVIP), Netherlands Food and Consumer Product Safety Authority (NVWA), Wageningen, the Netherlands; 5 European Center of Excellence for Vector Control, Rentokil Initial-Laboratorios Lokimica, Paterna, Valencia Spain; 6 Servei de Control de Mosquits del Consell Comarcal del Baix Llobregat, Barcelona, El Prat de Llobregat, Spain; 7 IRTA, Programa de Sanitat Animal, Centre de Recerca en Sanitat Animal (CReSA), Campus de la Universitat Autònoma de Barcelona (UAB), Barcelona, Spain; 8 Centro Agricoltura Ambiente “Giorgio Nicoli” S.R.L. (CAA), Bologna, Crevalcore , Italy; 9 Centre for Ecological Research and Forestry Applications (CREAF), Cerdanyola del Vallès, Spain; 10 Institut Català de Recerca i Estudis Avançats (ICREA), Barcelona, Spain

**Keywords:** *Aedes albopictus*, Population structure, Microsatellites, Kinship, Points of entry

## Abstract

**Background:**

The Asian tiger mosquito, *Aedes albopictus*, is an aggressive invasive vector responsible for transmitting important arboviruses. Its global spread has been largely facilitated by human-mediated transport, especially through trade and road networks. Since its first detection in the Netherlands in 2005, repeated introductions have occurred via pathways such as lucky bamboo imports, used tires, aircraft, and ground traffic. Despite ongoing surveillance and elimination efforts, uncertainties remain about the origins, recurrence, and establishment potential of these introductions.

**Methods:**

We analyzed 200 *Ae. albopictus* specimens collected from 21 locations in the Netherlands from 2014 to 2023, including detections in residential areas and points of entry (PoEs). Samples were genotyped at 19 specific microsatellite loci. Genetic diversity and kinship were studied to better understand genetic structure, relatedness between and within locations, and local overwintering. We also determined the presence of *Wolbachia* endosymbiont strains in the specimens by sequencing *Wolbachia* markers.

**Results:**

Genetic structure and kinship analyses revealed multiple independent introductions, genetic diversity among sites, and evidence of local overwintering at both residential and PoE locations, including used tire storage sites. Close-kin relationships were identified in 63 specimen dyads. Among these, one dyad confirmed overwintering at a used tire storage facility, four indicated kinship within a residential area, and two between two locations. Genetic assignment results also highlighted successful elimination of the species in one Dutch residential area. A total of 16 Dutch locations (76.19%) tested positive for the presence of *Wolbachia*. Overall, 48.86% specimens analyzed tested positive for at least one strain, and 35 close-kin dyads showed complete concordance in *Wolbachia* infection status.

**Conclusions:**

Our findings highlight the complex invasion dynamics of *Ae. albopictus* in the Netherlands. Our results demonstrate that microsatellite analysis, combined with kinship assessment, is an efficient approach for investigating kinship among individuals within and between urban areas and PoEs, providing evidence of local overwintering, and assessing the genetic structure of *Ae. albopictus* at introduction sites. The widespread presence of *Wolbachia*, which is known to reduce mitochondrial diversity, suggests that mitochondrial DNA (mtDNA)-based population analyses may be limited for the species.

**Graphical Abstract:**

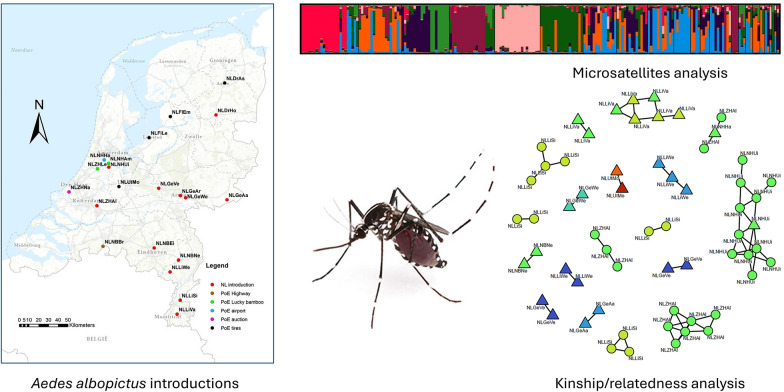

**Supplementary Information:**

The online version contains supplementary material available at 10.1186/s13071-026-07415-z.

## Background

The Asian tiger mosquito, *Aedes albopictus* (Diptera: Culicidae), is an invasive species known for its aggressive biting behavior, which causes significant discomfort [1]. Laboratory studies have demonstrated that it can transmit over 22 different viruses [2]. Its native range is believed to include Japan, China, northern India, and parts of Southeast Asia [3]. During the seventeenth and eighteenth centuries, *Ae. albopictus* spread to several islands across the Pacific and Indian Oceans [4]. Owing to global trade and human-facilitated movement, the species has since expanded to all continents except Antarctica [5].

The first established populations of *Ae. albopictus* in Europe were recorded in Albania in 1979 [6]. In Italy, where the species was first detected in the northern regions in 1990 [7], the population is thought to have dual origins: one linked to international trade involving used tires imported from the eastern coast of the USA, and another through gradual spread of populations in Albania and Greece that had originated in East Asia [8, 9]. The species’ presence in Spain, France, Switzerland, and, more recently, parts of Germany, has been attributed to its spread from Italy along road networks and highways [10–12]. Human transportation appears to be *Ae. albopictus’* most common dispersal mode [13], with ground vehicles serving as the most important drivers of its spread at national and continental scales in Europe [11, 14].

In the Netherlands, the Asian tiger mosquito was detected for the first time in 2005 at the premises of companies importing and growing lucky bamboo plants [15]. Since then, multiple introduction events via various pathways have been recorded, and the species has been detected annually through exotic mosquito surveillance efforts [16] as well as through reports from residents to the Dutch Centre for Monitoring of Vectors (CMV). Besides lucky bamboo importers, *Ae. albopictus* has been consistently found at storage facilities of used tire companies, and sporadically at other points of entry (PoEs) such as airports and flower auction sites [17], as well as at parking lots along major highways and residential urban areas. Dutch policy aims to delay the establishment of invasive *Aedes* mosquitoes as long as possible. Active monitoring and elimination campaigns are executed to prevent the species from becoming permanently established. Early detections at monitored locations such as PoEs and tire companies’ premises included in CMV’s active surveillance can usually be swiftly eradicated. However, detections in residential urban areas, reported by residents, cannot be anticipated and often require labor-intensive door-to-door elimination efforts. The sources of these residential introductions remain uncertain, but they are hypothesized to result almost entirely from accidental transport by private individuals via ground traffic from other European regions where the species is established.

In our previous study, we sequenced the complete mitogenomes (mitochondrial DNA [mtDNA]) of 219 *Ae. albopictus* specimens, along with 35 previously published mitogenomes, and generated a Nextstrain build [16]. The goal was to identify linkages between mtDNA haplotypes and the sources or possible pathways of introduction. Results revealed low mitogenomic diversity among the *Ae. albopictus* specimens. Genomic variation among specimens from the Netherlands was largely confined to a single major clade, which is hypothesized to correspond to the globally invasive strain of the species. Our results showed no clear links, making it difficult to trace origin of new introductions or assess mosquito overwintering or the success of the elimination campaigns [16]. Furthermore, almost identical mtDNA sequences were obtained in individuals from different locations, suggesting the possible presence of endosymbionts, such as *Wolbachia* sp., that could introduce a selective sweep resulting in low mitogenomic variation [12, 18]. Although the mitogenome is often used as a population marker owing to its maternal inheritance and steady mutation rate, it has limitations that can hamper the resolution in some species such as the fact that mtDNA may not be strictly clonal due to events such as recombination and heteroplasmy, or selective sweeps due to the presence of *Wolbachia* sp. [19].

The aim of the current study was to evaluate nuclear DNA variation, specifically using microsatellite markers, in the samples previously analyzed for mtDNA [16] and in additional samples, with the ultimate goal of establishing stronger connections to geographical origins and introduction pathways. Additionally, using kinship analysis, we intended to identify kin relationships between locations and across years within the same location, to help clarify whether populations are overwintering locally or appearing from new introductions. Finally, we aimed to determine the presence of *Wolbachia*, a maternally transmitted symbiont, to determine whether it could be the cause of the low mtDNA diversity observed in our previous study. Better understanding of the origins, pathways, and individual relationships of these introductions can enhance risk-based surveillance (active and passive) and control strategies for *Ae. albopictus* in the Netherlands.

## Methods

### Collection of mosquito specimens

**Fig. 1:**
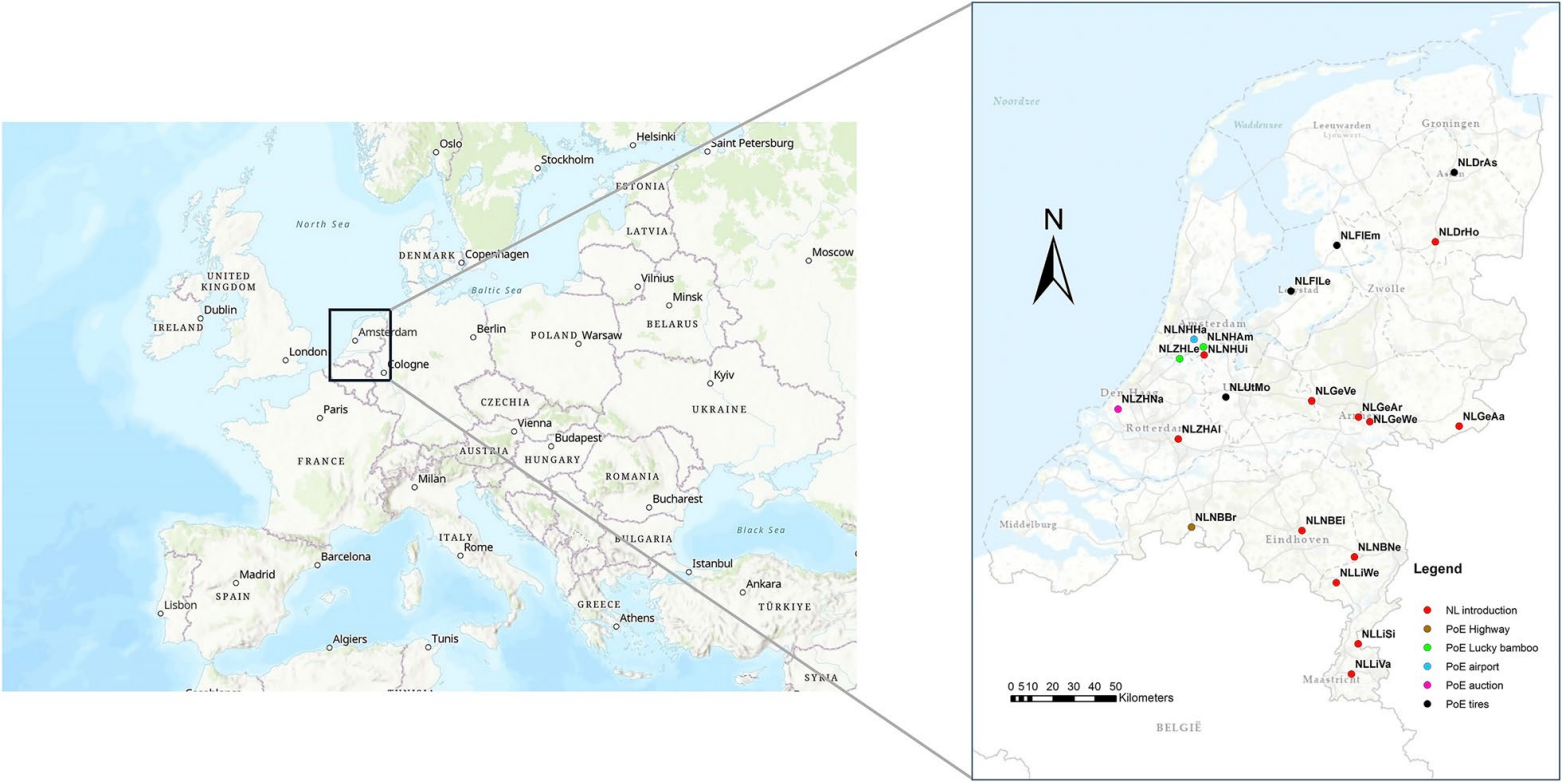
Map representing the 21 locations of *Ae. albopictus* collected between 2014 and 2023 in the Netherlands. Dutch specimens were collected from urban locations with unknown introduction pathways (Netherlands [NL] introductions) or found during surveillance at PoEs such as used tire companies (PoE tires), lucky bamboo importers (PoE lucky bamboo), parking lots alongside highways (PoE highway), airports (PoE airport), or flower auctions (PoE auctions). Details on sampled locations, which are identified by a unique six-letter abbreviation, year of collection, and number of samples per location, are presented in Table 1. Map created in ArcGIS Pro (ESRI) using World Topographic Map basemap

We analyzed 200 *Ae. albopictus* specimens collected in the Netherlands from 2014 to 2023. The specimens were collected at 21 locations (Fig. 1) and included adult female mosquitoes (*n* = 145), larvae (*n* = 50), and eggs (*n* = 5) (Table 1). Dutch specimens were collected from urban residential areas or found during surveillance at PoEs such as used tire companies, lucky bamboo importing companies, parking lots alongside highways, airports, or flower auctions. Except for one location (NLLiVa; Table 1), the mosquito populations at all other collection sites had been eliminated through intensive mosquito control by the time of article submission (November 2025). This sample set includes 161 specimens from locations of our previous mtDNA analysis [16] (Supplementary Material 1: Supplementary Table S1). In addition to the 200 specimens from the Netherlands, the sample set was expanded with a total of 19 specimens from Spain (*n* = 5), Italy (*n* = 6), and China (*n* = 8) (Table 1). Specimens were captured using different methods, including adult traps, gravid traps, larval sampling, and egg collection using ovitraps (Table 1). Larval specimens were preserved in 70% ethanol, and adults and eggs were preserved dry. All collected specimens were stored at −20 °C for further analysis.

**Table 1:** Metadata for the 24 *Aedes albopictus* sampling locations included in this study obtained from the Netherlands, Italy, Spain, and China from 2014 to 2023. For each population, information on the location, life stage, and collection method is provided. The table reports the total number of samples analyzed and the percentage and number (*N*) of samples with at least 15 positive loci. Additionally, the introduction pathway is listed for each population. The location code corresponds to the unique identifier used in all analyses

Country	Life stage	Region/province	Municipality/city	Location code	Pathway	Number of samples	Collection year(s)	Collection method	Percentage of samples with minimum 15 positive loci (*N*)
China	Adult	Guangdong	Guangzhou	CHGuGu	Reference	8	2016	Reared adults from eggs	87.50% (7)
Spain	Adult	Cataluña	Barcelona	ESCaBa	Reference	2	2016	Mouth aspirator	100.00% (2)
		Valencia	Valencia	ESVaVa	Reference	3	2016	BG-Sentinel	100.00% (3)
Italy		Emilia Romagna	Copparo	ITERCo	Reference	3	2016	CDC trap	66.67% (2)
		Emilia Romagna	Ravenna	ITERRa	Reference	3	2016	CDC trap	66.67% (2)
Netherlands		Noord-Brabant	Eindhoven	NLNBEi^b^	NL introduction	1	2018	Hand net	100.00% (1)
			Neerkant	NLNBNe^b^	NL introduction	3	2019	Hand net, BG-Sentinel, BG-GAT	100.00% (3)
		Nord Holland	Uithoorn	NLNHUi^a^	NL introduction	3	2019	BG-Sentinel	100.00% (3)
			Amstelveen	NLNHAm^b^	PoE Lucky bamboo	1	2015	Mosquito magnet trap	100.00% (1)
			Haarlemmermeer	NLNHHa^b^	PoE airport	4	2018, 2019	BG-Mosquitaire	100.00% (4)
		Limburg	Sittard	NLLiSi^a^	NL introduction	3	2020	BG-Sentinel	100.00% (3)
			Valkenburg	NLLiVa	NL introduction	28	2019, 2020, 2023	BG-Sentinel	85.71% (24)
			Weert	NLLiWe	NL introduction	13	2016, 2017, 2018	BG-Sentinel	100.00% (13)
		Zuid-Holland	Alblasserdam	NLZHAl^a^	NL introduction	2	2019	BG-Sentinel	100.00% (2)
			Naaldwijk	NLZHNa^b^	PoE flower auction	2	2018, 2019	BG-Mosquitaire	100.00% (2)
			Leimuiden	NLZHLe^b^	PoE lucky bamboo	2	2016	Mosquito magnet trap, BG-Sentinel	100.00% (2)
		Drenthe	Assen	NLDrAs	PoE tires	15	2015, 2022, 2023	BG-Sentinel	93.33% (14)
			Hoogeveen	NLDrHo^b^	NL introduction	3	2019	BG-Sentinel	100.00% (3)
		Flevoland	Emmeloord	NLFlEm^b^	PoE tires	3	2014, 2016	BG-Sentinel	100.00% (3)
			Lelystad	NLFlLe^b^	PoE tires	1	2016	BG-Sentinel	100.00% (1)
		Gelderland	Aalten	NLGeAa	NL introduction	27	2017, 2023	BG-Sentinel	66.67% (18)
			Arnhem	NLGeAr^b^	NL introduction	1	2018	Hand net	100.00% (1)
			Veenendaal	NLGeVe	NL introduction	18	2016	BG-Sentinel	100.00% (18)
			Westervoort	NLGeWe^b^	NL introduction	3	2018	Hand net, BG-Sentinel	100.00% (3)
		Utrecht	Montfoort	NLUtMo	PoE tires	12	2022, 2023	BG-Sentinel	91.67% (11)
	Larvae	Noord-Holland	Uithoorn	NLNHUi^a^	NL introduction	16	2019	Larval sampling	100.00% (16)
		Limburg	Sittard	NLLiSi^a^	NL introduction	17	2020	Larval sampling	100.00% (17)
		Zuid-Holland	Alblasserdam	NLZHAl^a^	NL introduction	17	2019	Larval sampling	100.00% (17)
	Egg	Noord-Brabant	Breda	NLNBBr	PoE highway	5	2020	Ovitrap	80.00% (4)

^a^Locations with samples representing different life stages (e.g., eggs, larvae, adults)

^b^Locations with less than five samples

### DNA extraction

Analysis was performed on individual, complete specimens, maintaining intact heads for all adult mosquitoes without prior rehydration. Two different methods of DNA extraction were used. Genomic DNA was extracted from 63 samples using the High Pure PCR Template Preparation Kit (Roche, Switzerland), and from 156 samples using the automated KingFisher Flex System (Thermo Fisher Scientific, MA, USA) with the QuickPick^™^ SML gDNA Kit (Bio-Nobile, Finland), following the manufacturers’ protocols. Prior to DNA extraction, individual specimens (adults, larvae, and eggs) were ground with a sterile plastic pestle with Proteinase K and lysis buffer from the DNA extraction kit. The DNA was stored at −20 °C for further analysis.

### Microsatellite screening and analysis

Samples were genotyped at 19 microsatellite loci specific for *Ae. albopictus* [20–22] combined in three multiplexes (Supplementary Material 1: Supplementary Table S2). Multiplex polymerase chain reaction (PCR) amplifications were carried out in 11 µL of reaction volume, containing 5.5 µL of Qiagen Multiplex PCR Master Mix, 1.0 µL of genomic DNA, 1.0 µL of Q-Solution, and variable volumes of primer mix and nuclease-free water. Thermocycling conditions included an initial denaturation at 95 °C for 15 min, followed by 35 cycles at 94 °C for 30 s, 58 °C for 90 s, and 72 °C for 1 min, with a final extension at 72 °C for 30 min. PCR products were stained with GelRed (Biotium), separated on 1.2% agarose gels, and visualized under ultraviolet (UV) light. Genotyping of the PCR products was performed by Secugen S.L. (Madrid, Spain) on a 48-capillary 3730xl DNA Analyzer (Applied Biosystems, Thermo Fisher Scientific, Waltham, MA, USA). Fragments were sized with GeneScan 500 Liz internal size standard (Applied Biosystems, Waltham MA, USA) and binned using Geneious Prime version 2024.0.5 (Biomatters, New Zealand) microsatellite plugin [23]. Microsatellite loci that exhibited an excessive amount of missing data (> 25%), as well as individuals that could not be reliably scored for at least 15 loci, were excluded from further analyses.

Given the limited sample sizes, individuals were analyzed as “sampling groups” rather than true populations, reflecting an operational grouping for genetic diversity and structure analyses. The presence of potential scoring errors, stuttering, large allele dropout, and null alleles was tested using Micro-Checker version 2.2.3 [24]. The frequency of null alleles for each locus and sampling group was further investigated using the expectation maximization algorithm implemented in FreeNA [25]. The same program was used to calculate global fixation index (FST) values corrected for null alleles following the excluding null alleles (ENA) correction method. Bootstrap 95% confidence intervals (CI) were calculated using 1000 replicates over loci.

To estimate possible genotyping errors, we used the software Colony [26, 27]. Colony implements a maximum likelihood method for estimating both parentage/sibship and genotyping error rates at each locus. The software was run with the null allele frequencies computed with FreeNA as allelic dropout rate, and the rate of other kinds of genotyping errors was set to 0.01 at all loci, following Mokhtar-Jamai et al., 2013 [28].

Genetic diversity at sampling groups was assessed in GenAlEx version 6.5, using the average number of alleles (Na), effective number of alleles (Ne), Shannon’s diversity index (I), observed heterozygosity (Ho), expected heterozygosity (He), and number of private alleles [29]. Private alleles represent genetic variants found exclusively in a single sampling group and therefore serve as useful indicators of differentiation, potential independent introduction events, or restricted gene flow. Sampling groups comprising fewer than five individuals were excluded from all genetic analyses except private allele calculations to minimize the risk of biased estimates. The following standard genetic parameters were also computed per locus using GenAlEx: total gene diversity (Ht), expected heterozygosity (He), and observed heterozygosity (Ho), inbreeding coefficient within subpopulations (Fis), total inbreeding coefficient (Fit), genetic differentiation among populations (Fst), and gene flow (Nm).

Additionally, to assess genetic differentiation and similarity among *Ae. albopictus* from different locations, pairwise Nei’s genetic distances (D) and Nei’s genetic identity (I) [30] were calculated for locations with more than five specimens. The resulting matrices were organized into a combined table, and a phylogenetic tree was constructed in Poptree2 [31] on the basis of Nei’s genetic distances, using the neighbor-joining (NJ) algorithm method with 1000 bootstrap replicates. A principal coordinates analysis (PCoA) was performed to explore genetic relationships among the 24 *Ae. albopictus* locations, including the 3 locations from Italy, Spain, and China. Codominant genotypic distance matrices were calculated on the basis of microsatellite data and subsequently used to construct principal coordinate axes using GenAlEx. All distance calculations and coordinate extractions were conducted using default settings. Genetic assignment to sampling locations was performed using the model-based clustering approach implemented in STRUCTURE version 2.3.4 [32]. The number of assumed populations (K) ranged from 1 to 24. For each K, ten independent runs were performed, with a burn-in of 100,000 iterations followed by 500,000 Markov chain Monte Carlo (MCMC) repetitions. StructureSelector [33] was used to visualize the resulting genetic clusters and to identify the most likely number of populations (optimal K) on the basis of model selection criteria. For NJ and STRUCTURE analyses, and owing to the small number of specimens collected from two locations in Spain and two locations in Italy, all samples from each of these countries were pooled into a single sampling group.

GenePlot [34] was used to investigate fine-scale population structure at locations resampled over multiple years to assess the effectiveness of eradication programs (including only localities with more than five individuals). This allowed us to determine whether re-invaded sites were due to surviving individuals or new independent colonizations. Analyses were performed using Baudouin and Lebrun’s (2001) method [35], which is robust to rare alleles and therefore mitigates sampling bias in small populations.

### Kinship analysis

To infer kinship between all individual pairs, we used both ML-RELATE and CKMRsim [36, 37] for comparison. Kinship was classified into four categories: parent-offspring (PO), full-siblings (FS), half-siblings (HS), and unrelated (U). As for ML-RELATE, we set confidence intervals at 0.95 and applied 10,000 randomizations. As for CKMRsim, we simulated 200,000 replicates per relationship category to generate likelihood scores for kinship inference. Error rates for the different kinship assignments were calculated. We set false-positive rates to be close to a hundredth of the inverse of the number of comparisons (200 × 199), as recommended in the CKMRsim manual. We used standard network visualization tools in R packages “tidyverse,” “igraph,” and “ggnetwork” [38, 39] to build a mosquito network depicting all significant close-kin dyads.

### *Wolbachia* genotyping

To detect *Wolbachia* in *Ae. albopictus*, the generic primers 81F and 691R (Supplementary Material 1: Supplementary Table S3) were used to amplify a 601–625-nucleotide (nt) fragment (depending on the strain) of the *wsp* gene, which encodes the outer surface protein precursor of *Wolbachia* spp. [40, 41]. When *Wolbachia* was detected, strain-specific primers were used for these samples. The primer pair 328F and 691R was used for the detection of *Wolbachia* strain A (wAlbA), while the primer pair 183F and 691R targeted *Wolbachia* strain B (wAlbB) [41]. All PCRs were conducted in a total volume of 25 µL, containing 1 U of DreamTaq Hot Start DNA Polymerase (Thermo Fisher Scientific Inc., Waltham, MA, USA), 1× DreamTaq Buffer, 200 µM of each dNTP, 0.2 µM of each primer, 2 µL of template DNA, and Milli-Q water. Amplification conditions for all primer combinations included an initial denaturation at 95 °C for 3 min, followed by 30 cycles of 95 °C for 30 s, 55 °C for 30 s, and 72 °C for 1 min, with a final extension at 72 °C for 15 min. PCR products were purified using a NucleoFast 96 PCR Plate (Macherey–Nagel) and sequenced in both directions on an ABI 3730 capillary sequencer (Secugen, Madrid, Spain). To validate results, two PCR replicates were performed for each sample, following the approach described in [42]. A third replicate was performed for samples that showed inconsistent results between the first two replicates. Trace files were imported and analyzed in Geneious Prime.

## Results

### Population genetic statistics

Of the 19 microsatellite loci analyzed, 1 locus (i.e., alb-tri-46) failed to amplify consistently, with a success rate below 75%, and was therefore excluded from further analysis. Additionally, out of 219 specimens initially tested, 19 yielded results for fewer than 15 loci and were subsequently excluded. As a result, the final dataset comprised 200 *Ae. albopictus* specimens from 4 countries and 24 locations (Table 1).

Micro-Checker did not find evidence of scoring errors, stuttering, or large allele dropout. Potential presence of null alleles was found at nine loci (i.e., Alb-tri-3, Alb-tri-41, Alb-tri-45, Aealbmic4, Aealbmic5, Aealbmic7, Aealbmic11, Aealbmic12, and Aealbmic13). According to FreeNA, mean null allele frequency across all loci and sampling locations was 0.073, ranging from 0.00 to 0.40. Nonetheless, global FST values with and without correcting for null alleles were 0.113 and 0.115, respectively, and had overlapping 95% confidence intervals (CI 0.098–0.138 for FST using ENA and 0.095–0.137 for FST not using ENA), indicating that the impact of null alleles is negligible and likely not affecting kinship results. Genotyping errors as assessed in Colony are presented in Supplementary Material 1: Supplementary Table S4.

A total of 16 private alleles were identified across seven loci, representing genetic variants found exclusively in a single specific location (Supplementary Material 1: Supplementary Table S5). These alleles occurred at relatively low frequencies. Notably, the Chinese (CHGuGu) and Italian (IT) sampling groups carried one private allele at locus Alb-tri-18 each, with frequencies of 0.14 and 0.25, respectively. Several private alleles were also detected within Dutch locations, particularly in the used tire location NLDrAs, the residential area NLGeVe, and the lucky bamboo importer NLNHAm. Genetic diversity and distance results at microsatellite loci assessed in GenAlEx are presented in Supplementary Material 1: Supplementary Text 1.

### Genetic structure analyses

**Fig. 2:**
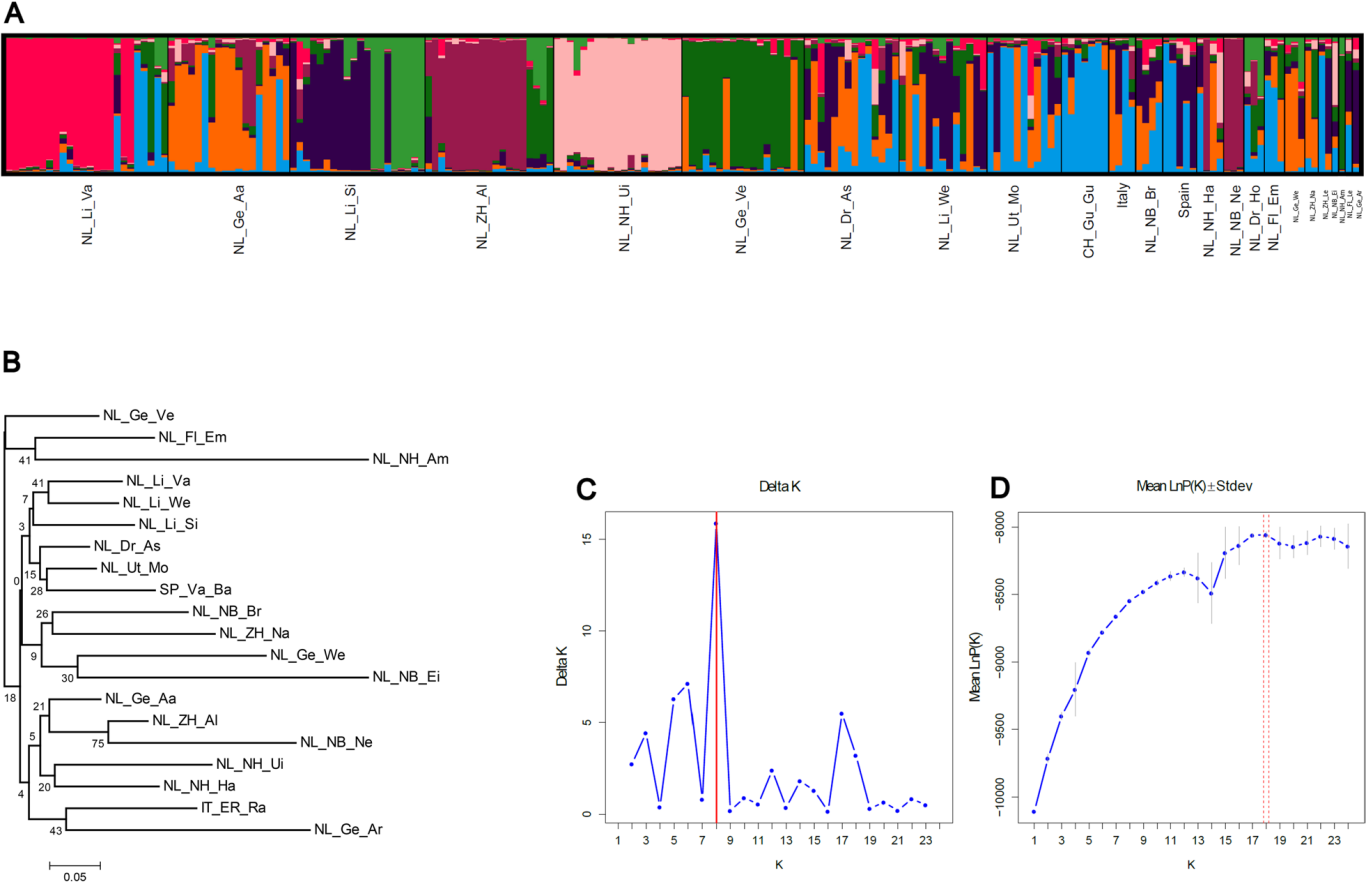
**A** Plot of the model-based clustering results for *K* = 8 implemented in STRUCTURE. Each *Ae. albopictus* individual is represented by a vertical bar corresponding to the sum of assignment probabilities to the K cluster (*K *= 8). Black lines separate populations. Locality abbreviations are available in Table 1. **B** NJ tree, calculated on the basis of Da distance, with four populations discarded from analysis owing to excessive amount of missing data. **C** Delta K, **D** Mean LnP(K) + standard deviation (SD)

The PCoA (Supplementary Material 1: Supplementary Fig. S1) revealed almost no discernible genetic structure among the studied locations of *Ae. albopictus*. The first two axes explained 34.09% of the total genetic variation (PCoA1: 20.41%; PCoA2: 13.68%). Only the Dutch location NLNHUi, whose individuals were collected in a residential area, showed a marked genetic differentiation from the other populations, forming a distinct cluster along axis 2. The NJ tree (Fig. 2B) showed several distinct clusters, indicating noticeable genetic diversity across the sampled locations, with some locations showing very close relationships (e.g., NLLiWe and NLLiSi), while others appeared more distantly related (e.g., NLZHAI and NLNBNe).

STRUCTURE-based clustering analysis indicated an optimal K value of 8, representing the best-supported number of ancestral clusters (Fig. 2C). The results showed high genetic variability, both between and within sampled locations as well as clear evidence of admixture in most of the sampling groups analyzed (Fig. 2A). Genetically admixed sampling groups include the used tire sites NLDrAs, NLLiWe, and NLUtMo, the highway parking lot NLNBBr, and the residential location NLGeAa. Additionally, a clear dominance of two ancestral lineages was observed in Dutch residential areas NLLiVa, NLLiSi, and NLZHAl. Relative dominance of a single ancestral lineage was found in Dutch residential locations NLNHUi, NLGeVe, and the Chinese location CHGuGu. The ancestral lineage dominant in Dutch residential location NLNHUi was rarely present in other locations included in the study. A similar dominance of one ancestral lineage (only in three individuals) was also observed at residential location NLNBNe. Notably, the same lineage was highly dominant at the residential location NLZHAl. In contrast, the four specimens collected at airport location NLNHHa exhibited high diversity and low similarity.

GenePlot was used to investigate fine-scale population structure at the five localities that were resampled over multiple years and included more than five individuals: NLLiVa, NLGeAa, NLDrAs, NLLiWe, and NLUtMo. At residential site NLLiVa, individuals sampled in 2019 and 2020 were assigned to the same genetic cluster, whereas individuals collected in 2023 were assigned to a different cluster (Supplementary Material 1: Supplementary Fig. S2), suggesting that the 2023 specimens likely represent a new, independent introduction. Consistent with these findings, STRUCTURE results (Fig. 2A) showed a sharp genetic break between the early and late sampling periods. A similar pattern was observed at location NLGeAa, where individuals sampled in 2023 clustered separately from all but one individual collected in 2017. In contrast, results from the remaining locations showed no clear cluster separation, preventing a reliable assessment of whether re-invaded sites originated from locally surviving individuals or from new, independent introductions of the species.

### Kinship analysis

The results of kinship analysis based on both ML-RELATE and CKMRsim were concordant. However, the calculated error rates indicated low false-positive rates but very high false-negative rates, to the extent that parent–offspring dyads could not be reliably distinguished from full- or half-sibling pairs (Supplementary Material 1: Supplementary Table S6). We therefore collapsed PO/FS/HS categories into a single first-degree kinship class and compared these with non-first-degree unrelated dyads. Although this prevents confident discrimination among specific kinship types, the low false-positive rate indicates that inferred kin dyads likely represent true relatedness with high certainty.

**Fig. 3:**
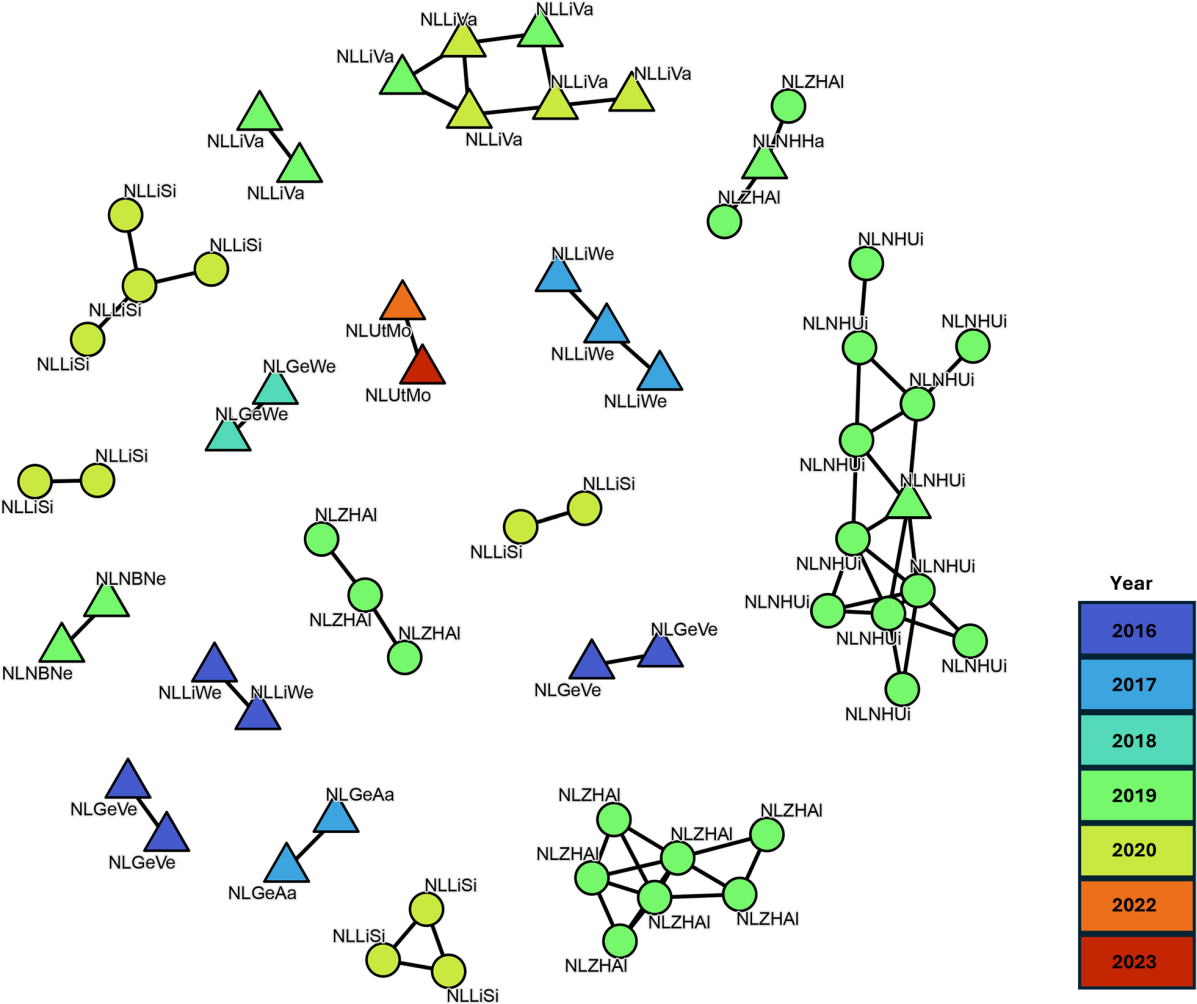
Mosquito network showing all *Ae. albopictus* mosquito pairs displaying first-degree kinship. Nodes represent mosquito specimens, and lines link pairs of related specimens. Symbols represent specimen developmental stage: triangle = adult, circle = larvae. Locations are identified by a unique six-letter abbreviation. Locality abbreviations are provided in Table 1

The mosquito network comprised 63 close-kin dyads organized into 18 groups of related mosquitoes (i.e., mosquitoes showing first-degree kinship), including one relatively larger group from the Dutch location NLNHUi, alongside two other important groups from residential areas NLZHAl and NLLiVa and 15 smaller, isolated groups (Fig. 3). Mosquitoes in the network are depicted on the basis of sampling location, collection year, and life stage (adult, larvae, or egg) to assess potential dispersal and overwintering. In general, groups were formed by mosquitoes belonging to the same location: Of the 24 studied locations, within-location individual kinship was detected in 10 Dutch locations, and between-location kinship was detected in 1 dyad only.

Four groups were found for the location NLLiSi with related specimens, and two groups for locations NLLiVa and NLGeVe. Interestingly, five pairs of mosquitoes from the same locations, one from NLUtMo (used tire location) and four from NLLiVa (urban residential location), whose individuals were collected in different years, showed kinship (Fig. 3). This suggests evidence of local overwintering within both sampling locations. Kinship was also found between specimens belonging to two different locations. One female adult collected at the cargo facilities at Schiphol Airport NLNHHa in 2019 showed a close-kin relationship with two larval specimens collected at location NLZHAl the same year (Supplementary Material 1: Supplementary Table S7).

In total, 21 close-kin dyads were found between individuals from the same location (NLNHUi) collected in 2019. One specimen was an adult female captured with a mosquito trap and showed close-kin relationship with five larval specimens. Interestingly, these larval specimens showed close-kin relation with other larval specimens collected at other breeding sites at the same location.

Close-kin between adult female pairs was detected at nine locations (Supplementary Material 1: Supplementary Table S7). At location NLGeWe, the first notified adult female in 2018 was found having close-kin with an adult female collected in a trap after 1 week. At location NLLiWe, three adult female close-kin dyads were collected in a trap in 2016 and 2017, with one from 2017 having close-kin relation with an adult female collected the same year in another trap situated 200 m away. Local overwintering at residential areas was also detected as four close-kin dyads were identified at NLLiVa between adult specimens captured in 2019 and 2020. Overwintering at PoE was also detected, as one close-kin dyad was identified at NLUtMo (used tire location) between two adult specimens captured in 2022 and 2023.

### *Wolbachia* genotyping

A total of 219 *Ae. albopictus* specimens were screened for the presence of *Wolbachia* symbiont of *Ae. albopictus* (Table 2). Out of these, 107 (48.86%) specimens tested positive for at least one *Ae. albopictus* *Wolbachia* strain on the basis of PCR amplification and subsequent sequencing. *Wolbachia* strain A (wAlbA) was detected in 87 individuals, of which 83 showed coinfection with strain B (wAlbB). *Wolbachia* strain B (wAlbB) was detected in a total of 103 individuals. All sequences obtained from both *Wolbachia* endosymbiont strains were identical to each other. In addition, 16 of the 21 Dutch locations (79.19%) tested positive for the presence of *Wolbachia*. No *Wolbachia* was detected in the Dutch populations NLNBBr (*n* = 5 eggs), NLNBNe (*n* = 3 adults), NLZHLe (*n* = 2 adults), NLNHAm (*n* = 1 adult), and NLFLLe (*n* = 1 adult), and in the samples from China (CHGuGu).

**Table 2:** Infection rate of *Ae. albopictus* specimens with *Wolbachia* strain A (wAlbA), *Wolbachia* strain B (wAlbB), and coinfection percentage of wAlbA and wAlbB. The last column shows the percentage of specimens within the locations where no *Wolbachia* strain was detected. The locations are ordered on the basis of the number of samples used in the analysis

Location name	Location number	Stage	Number of samples	Only wAlbA (%)	Only wAlbB (%)	wAlbA + wAlbB (%)	No *Wolbachia* (%)
NLLiVa	1	Adult	28	3.57	17.86	42.86	35.71
NLGeAa	2	Adult	27	0.00	7.41	37.04	55.56
NLLiSi	3	Larva + adult	20	0.00	5.00	25.00	70.00
NLZHAl	4	Larva + adult	19	5.26	0.00	52.63	42.11
NLNHUi	5	Larva + adult	19	5.26	15.79	42.11	36.84
NLGeVe	6	Adult	18	0.00	16.67	72.22	11.11
NLDrAs	7	Adult	15	6.67	0.00	20.00	73.33
NLLiWe	8	Adult	13	0.00	15.38	76.92	7.69
NLUtMo	9	Adult	12	0.00	0.00	8.33	91.67
CHGuGu	10	Adult	8	0.00	0.00	0.00	100.00
ITERRa + ITERCo	11	Adult	6	0.00	50.00	0.00	50.00
NLNBBr	12	egg	5	0.00	0.00	0.00	100.00
ESCaBa + ESVaVa	13	Adult	5	0.00	0.00	0.00	100.00
NLNHHa	14	Adult	4	0.00	0.00	50.00	50.00
NLNBNe	15	Adult	3	0.00	0.00	0.00	100.00
NLDrHo	16	Adult	3	0.00	0.00	66.67	33.33
NLFlEm	17	Adult	3	0.00	0.00	33.33	66.67
NLGeWe	18	Adult	3	0.00	33.33	66.67	0.00
NLZHNa	19	Adult	2	0.00	0.00	100.00	0.00
NLZHLe	20	Adult	2	0.00	0.00	0.00	100.00
NLNBEi	21	Adult	1	0.00	0.00	100.00	0.00
NLNHAm	22	Adult	1	0.00	0.00	0.00	100.00
NLFLLe	23	Adult	1	0.00	0.00	0.00	100.00
NLGeAr	24	Adult	1	0.00	0.00	100.00	0.00

Results on *Wolbachia* infection status and strain identity for all inferred close-kin dyads are available in Supplementary Material 1: Supplementary Table S7. Among the 63 close-kin dyads, 35 (55.55%) showed complete concordance in *Wolbachia* status (same strain combination or shared absence). Importantly, we did not observe any dyad in which the two individuals carried clearly different strains (e.g., strain A versus strain B). The remaining dyads consisted of combinations in which one individual was infected and the other uninfected (e.g., A/absence, B/absence, or AB/absence; 34.9%) and considered also as potentially concordant.

Representative sequences of *Wolbachia* endosymbiont of *Ae. albopictus* from this study were deposited in GenBank under accession nos. wAlbA PX560811–PX560827 and wAlbB PX569349–PX569367.

## Discussion

The genetic patterns observed in *Ae. albopictus* across Dutch sampling locations provide insights into introduction dynamics and local persistence. The relatively high genetic diversity among locations suggests multiple independent introduction events, with international used tire import facilities likely acting as important entry points for diverse lineages. At the same time, findings indicate overwintering and persistence at specific locations. Close-kin relationship between mosquitoes sampled in different years at one used tire facility and at one residential area, together with the identification of 63 close-kin relationships (61 within locations and 2 between two locations), demonstrate strong location-associated relatedness and temporal continuity. GenePlot analyses further discriminate individuals from separate introduction events, illustrating the success of elimination campaigns in interrupting certain lineages.

### Genetic diversity of *Aedes albopictus* sampling locations

The observed genetic diversity among *Ae. albopictus* sampling locations in our study suggests multiple introduction events at various Dutch locations, accompanied by diverse demographic histories. Consistent with our findings, microsatellite analyses of ten populations in Germany [43] revealed heterogeneous population structures, indicating frequent independent introductions rather than continuous range expansion across the country. The genetic structure observed in the Netherlands confirms that *Ae. albopictus* individuals do not form a single homogeneous population but rather represent a mosaic of lineages shaped by complex invasion dynamics.

At PoEs such as used tire importers’ storage sites, highway parking lots, and airports, specimens of *Ae. albopictus* from diverse geographic origins are expected to be introduced at various times throughout the year. Genetic structure analyses at these PoE sites reveal high genetic variability both within and between locations, along with clear evidence of lineage admixture across most locations studied. This pattern is exemplified by the four specimens collected at Schiphol Airport (NLNHHa) over different years, which display high genetic divergence and low similarity in ancestral lineages, strongly suggesting multiple introductions via aircraft from distinct international airports. These findings align with previous studies on *Ae. aegypti* at the same location [44].

The observed genetic diversity within Dutch *Ae. albopictus* sampling groups suggests that some mosquito populations may already be locally established but undetected. Such populations could act as reservoirs for further dispersal and potential local adaptation, underscoring the importance of targeted mosquito control measures to contain their spread. Additionally, the occurrence of private alleles and relatively high heterozygosity in geographically distinct locations (e.g., NLDrAs, NLGeVe, and NLLiVa) indicates genetic differentiation among sites; however, this pattern may reflect a combination of factors, including independent introductions, post-introduction admixture, rapid genetic drift, local adaptation, incomplete or limited sampling size of source populations, or overwintering bottleneck.

### Overwintering and kinship

From the 63 dyads showing parental relationship, 5 dyads demonstrated overwintering at the location. At several used tire importers, repeated positive detections of *Ae. albopictus* eggs over consecutive years indicate either the arrival of new tire batches harboring eggs or successful overwintering at these sites [45]. Used tires likely provide favorable microhabitats for egg survival, larval development, and adult shelter, with factors such as storage conditions, tire type, and wind exposure influencing local microclimates. Supporting evidence for local overwintering includes close-kin observed between a mosquito pair sampled in different years at the NLUtMo used tire facility. Furthermore, the lowest Nei’s genetic distance (*D* = 0.08) and high genetic identity (*I* = 0.92) observed between the two used tire import locations NLDrAs and NLUtMo (Supplementary Material 1: Supplementary Text S1) indicate close genetic relationships, likely reflecting a recent shared import origin or trade between these companies.

Four adult mosquito dyads from the NLLiVa location, sampled in 2019 and 2020, demonstrated close-kin relation. Following targeted elimination campaigns and intensive surveillance, the population was considered eradicated in 2021. However, new specimens were detected in 2023 within the same municipality. The 2023 sample set also included specimens from this location where we hypothesized that populations might persist undetected despite prior elimination efforts. Individual assignment results demonstrate distinct genetic clusters between the 2023 collections and those obtained before the elimination campaigns in 2019 and 2020. Furthermore, no genetic kinship was detected between the 2019–2020 and 2023 specimens, indicating that the 2023 detections most likely represent a recent accidental introduction rather than a surviving, undetected local population following the elimination campaigns. This suggests that the campaigns were, in fact, effective.

### Implications of *Wolbachia* presence

Two *Wolbachia* strains were detected in the 219 specimens analyzed in this study, with identical DNA sequences observed among individuals for both strains. The presence of *Wolbachia* is known to reduce mitochondrial DNA (mtDNA) diversity—a phenomenon referred to as mtDNA homogenization or mitochondrial sweep [46], which effectively removes historical genetic variation.

Given the prevalence of this symbiont in our studied samples and the likely resulting mitochondrial DNA homogenization, mtDNA sequencing and visualization using platforms such as NextStrain may be less suitable for population structure analysis and origin tracing in *Ae. albopictus* [12]. However, mtDNA remains a useful marker for species with lower *Wolbachia* prevalence, and even for *Ae. albopictus*, as demonstrated by previous studies focused on introduction routes. Nonetheless, in our case, the greater genetic variability revealed by microsatellite markers suggests that these provide higher resolution and are more suitable for population genetic studies in this species.

We did not observe any dyad in which the two individuals carried clearly different *Wolbachia* strains (e.g., strain A versus strain B), and many dyads consisted of combinations in which one individual was infected and the other uninfected (e.g., A/absence, B/absence, or AB/absence; 34.9%). We consider these cases biologically compatible with concordance, as *Wolbachia* density can vary with temperature stress, host age, and methodological factors, potentially leading to false negatives during detection [47–49]. Therefore, the overall pattern suggests strain consistency within inferred close-kin pairs.

### Limitations of the study

The primary limitation of this study is the small number of specimens used to define possible populations. Microsatellite analyses typically recommend sampling between 25 and 30 individuals per sampling location for reliable genetic inference [50]. In our study, only the NLLiVa and NLGeAa locations included more than 20 specimens, while some locations were represented by a single individual (Table 1), which makes analyses particularly sensitive to stochastic effects. With such small sample sizes, the genetic variability of single samples can disproportionately influence summary statistics, potentially leading to biased estimates of diversity and misleading inferences about population structure or introduction history. Increasing sample sizes would enable more robust and representative comparisons between locations. Therefore, comparisons of genetic diversity among sampling locations should be interpreted with caution owing to the small and uneven sample sizes across sites. However, our aim was not to perform a population evolutionary study, but rather to characterize the genetic relatedness among individuals; therefore, different indices were used as descriptive means to facilitate comparisons among localities.

The low number of specimens is largely attributable to the success of mosquito elimination campaigns in the Netherlands. Following the detection of mosquitoes at these sites, rapid and effective control efforts eradicated the populations within a single season at most of the sites, preventing the collection of sufficient individuals for comprehensive genetic characterization and the establishment of stable populations. These elimination programs also hinder the development of long-term populations that might share common ancestral lineages. Owing to the prompt response by mosquito control teams after introductions, populations rarely develop beyond the offspring of the initial founding individuals, and our study therefore primarily included these pioneer offspring specimens. Furthermore, the success of elimination campaigns makes it impossible to repeat this study with specimens from these locations, as the populations no longer exist.

The absence of male *Ae. albopictus* specimens in the analysis may have reduced the accuracy of kinship assessments. Including males could improve the ability to detect stronger parent–offspring relationships. However, the adult trapping method that was used, relying on CO_2_ and skin-mimicking lures that specifically attract host-seeking females, resulted in a predominance of female specimens, which were therefore used in the study.

### Implications and future perspectives

The Netherlands has a policy that aims to prevent or delay the establishment of *Ae. albopictus* within the country for as long as possible. Intensive surveillance conducted annually at PoEs is crucial for detecting species introductions and initiating elimination programs promptly. Our results indicate that overwintering of *Ae. albopictus* occurred at two locations, including one PoE (used tire importer). This suggests that the mosquitoes found in a subsequent season either originated from dormant eggs that hatched after the winter or from eggs laid by females that emerged the previous year. The first scenario is possible as *Ae. albopictus* eggs can survive dry conditions for several months, facilitating overwintering in temperate zones [51, 52]. The latter scenario is also possible, although rapid control measures are taken at tire importers to eliminate local populations shortly after introduction [53]. However, the latter scenario is the most plausible in the residential area NLLiVa, where detections depend largely on residents’ notifications received by the Netherlands Food and Consumer Product Safety Authority (NVWA) service. Such notifications occur when residents experience nuisance from a small, aggressive, day-active black mosquito, a species not yet widely recognized, by which time a small population is typically already present in the area.

Genetic distance and identity analysis also revealed strong relationship between two geographically distant tire importers (NLDrAs and NLUtMo). While trade between these companies is known, this represents the first genetic result suggesting possible common sources of import. Interestingly, no genetic relatedness was detected between specimens collected at tire importers and those found in urban areas, which are presumed to have been accidentally introduced via private vehicles. If *Ae. albopictus* becomes established in municipalities where tire importers are located, this genetic approach could be applied to assess kinship between mosquitoes found within and around company premises and those detected elsewhere in the municipality. Such analysis could support efforts to attribute responsibility for the local spread of this invasive vector to specific commercial activities.

In the study by Sherpa et al. (2019) [13], it was demonstrated that the structure of human transportation networks has influenced the demographic history of *Ae. albopictus*, with northern and central Italy acting as primary dispersal hubs across Europe. Genetically diverse and admixed populations were shown to facilitate secondary introductions and disperse more widely compared with populations originating from a single source. In our study, we observed predominantly genetically admixed profiles at PoEs, likely reflecting the varied geographic origins of frequently imported specimens. These findings reinforce the importance of our ongoing efforts at PoE sites since 2010, aimed at preventing the establishment of potentially admixed populations that may possess a greater capacity for local spread.

*Aedes albopictus* is known to have a limited flight range, typically restricted to a few hundred meters [54]. Individuals of pairs showing overwintering at the NLLiVa location were found at a distance of more than 100 m from each other, and close-kin relationship was also detected between two individuals collected in 2017 from traps located 200 m apart at the NLLiWe site. Using kinship inference, we could estimate the active dispersal range of *Ae. albopictus* at these locations in newly invaded areas. Our findings also suggest that this approach could serve as a viable alternative to traditional mark–release–recapture methods for measuring active dispersal [20].

The introduction in the Netherlands of certain *Ae. albopictus* vector populations can bring undesirable traits, such as knockdown resistance mutations associated with pyrethroid insecticide resistance, highlighting the importance of identifying vector origins to assess the risk [55]. The introduction of specimens from regions with these resistant alleles raises concerns for vector control as these populations could be already resistant to authorized biocides. Furthermore, clarifying the origin of introductions and the relatedness among populations is crucial for assessing potential vector competence, as the vector competence of *Ae. albopictus* varies across geographical populations and virus species [56, 57].

Owing to the ongoing expansion of *Ae. albopictus* populations in Europe in terms of both geographic range and abundance, and the proximity of established populations in neighboring countries such as Belgium and Germany, it is expected that introductions into the Netherlands will become more frequent in the coming years. This raises concerns about the continued feasibility of successful elimination, as has been achieved to date. In the event of establishment, surveillance efforts should be intensified and include both spatial and temporal sampling to monitor changes in population structure, detect new introduction events, and track shifts in genetic diversity over time. Our study demonstrates the utility of genetic data in assessing genetic structure across sampling sites and relatedness among *Ae. albopictus* individuals. Our results also demonstrate that microsatellite analysis, combined with kinship assessment, is a robust approach for investigating the genetic structure of *Ae. albopictus* across locations, detecting evidence of local overwintering, and determining relatedness among individuals from different sites. In future work, we also plan to evaluate the use of a species-specific single-nucleotide polymorphism (SNP) chip to uncover fine-scale genetic patterns. This method has recently shown improved resolution for investigating population structure and invasion pathways, surpassing the capabilities of traditional mitochondrial DNA or microsatellite analyses [58].

## Conclusions

This study provides new insights into the genetic structure, introduction dynamics, and potential establishment of *Ae. albopictus* populations in the Netherlands. Using microsatellite markers and kinship analysis, we identified multiple independent introduction events, varying levels of genetic diversity across sites, and evidence of local overwintering at both residential areas and PoEs, including used tire importers. Genetically admixed profiles at PoEs suggest frequent introductions from diverse geographic sources, reinforcing the need to monitor high-risk sites. These findings underscore the complex invasion patterns of *Ae. albopictus* and the importance of continued surveillance and rapid response strategies to prevent local establishment. Results also suggest that the widespread presence of *Wolbachia* in half of the specimens analyzed likely plays a role in reducing mitochondrial diversity, thereby limiting the utility of mtDNA for fine-scale population structure studies. This highlights the need for nuclear markers such as microsatellites or SNP chips (single-nucleotide polymorphism arrays) for accurate genetic characterization. Given the expanding range of *Ae. albopictus* in Europe and its proximity to the Netherlands, introductions are likely to become more frequent. Maintaining intensive surveillance and incorporating high-resolution genomic tools will be essential to delay establishment and mitigate public health risks associated with this invasive mosquito species.

## Supplementary Information

Supplementary Material 1.

## Data Availability

The datasets supporting the conclusions of this article are included within the article and its additional files.

## Abbreviations

PoE: Point of entry
mtDNA: Mitochondrial DNA
CMV: Centre for Monitoring of Vectors (in the Netherlands)
PCoA: Principal coordinates analysis
NJ tree: Neighbor-joining tree
