# Supplementary material for: Evidence of kinship, overwintering, and Wolbachia presence in Aedes albopictus in urban areas and points of entry in the Netherlands
Source: Parasit Vectors. 2026 May 13;19:247. doi: 10.1186/s13071-026-07415-z (PMC13263923; doi:10.1186/s13071-026-07415-z)
Supplement: Supplementary file 1 — Supplementary Material 1. [file 13071_2026_7415_MOESM1_ESM.docx]

Supplementary information

**Supplementary Material 1: Supplementary Text S1.** **Genetic diversity and distance among sampling locations of *Aedes albopictus***

Total gene diversity (Ht) ranged from 0.34 (Alb-tri-21) to 0.82 (Aealbmic12), demonstrating that the markers differ considerably in the amount of genetic variation. Heterozygote deficiency, with Fis values > 0.3 was observed at 9 loci (Additional file 1: Table S8). Locus Alb-tri-21 showed the highest genetic differentiation (Fst = 0.42) and the lowest gene flow (Nm = 0.35), indicating pronounced population structure at this locus. In contrast, loci Alb-tri-41 and Alb-tri-33 showed lower differentiation, reflecting relatively higher gene flow (Fst = 0.09 and 0.12; Nm = 2.56 and 1.8, respectively).

Because genetic diversity provides insight into invasion history—such as whether populations originate from single or multiple introduction events—we assessed diversity for the 13 *Ae. albopictus* locations that met the minimum sample size of five individuals (Additional file 1: Table S8 and Table S9). We expected that sampling groups established through repeated or admixed introductions would exhibit higher diversity, whereas recently founded or isolated populations would show reduced variation. Genetic diversity varied notably among analysed sampled locations of *Ae. albopictus*. Dutch groups generally exhibited higher genetic diversity than those from southern Europe and China. Locations coded as NLLiWe, NLDrAs, and NLGeVe showed the highest allelic richness (Na > 4.6), effective alleles (Ne > 3.0), and expected heterozygosity values (He > 0.6). While specimens from location NLDrAs have been found introduced by import of used tires, the origin of the other two locations (NLLiWe and NLGeVe) is unknown but hypothesized to have been accidentally introduced by private individuals via ground traffic. These patterns reflect more genetically diverse or potentially admixed groups of *Ae. albopictus*. Specimens collected on a highway location (NLNBBr) and consisting of eggs collected in ovitraps, exhibited the lowest diversity (F=0.01) and the lowest percentage of polymorphic loci (83.3%).

Nevertheless, the Dutch sampling groups exhibited high genetic similarity among themselves, with the lowest Nei’s genetic distances observed between two used tire import locations NLDrAs and NLUtMo (D = 0.08). These pairs also showed correspondingly high genetic identity values (I = 0.92), reflecting close genetic relationships, possibly due to recent common ancestry or gene flow. Moreover, location NLDrAs exhibited the lowest Nei’s genetic distance to the residential area NLGeAa (D = 0.1), with respectively high genetic identity (I = 0.9) (Additional file 1: Table S9). These two pairs are geographically separated (approx. 130 Km) and characterised by a different introduction pathway (used tire import vs residential area; Fig. 1). In contrast, greater genetic distances were recorded between Dutch and non-Dutch locations. For example, NLLiVa showed relatively high genetic distances from IT (D = 0.58) and CHGuGu (D = 0.41), with low corresponding identity values (I = 0.56 and 0.66, respectively). Similarly, CHGuGu also displayed the highest distances to the residential area NLNHUi (D = 0.60).

**Supplementary Material 1: Supplementary Table S1.** Overview of samples and associated metadata, indicating the samples included in our previous mtDNA study (Ibañez-Justicia et al., 2022), and the samples analyzed in the current study. (*) Samples ultimately excluded from the microsatellite analyses because they did not meet the inclusion criteria.

| Sample ID | Location  number | Location code | Pathway | Year | Life stage | Used in mtDNA study | Used in current study |
| --- | --- | --- | --- | --- | --- | --- | --- |
| 32520617 | 1 | NLLiVa | NL introd | 2019 | Adult | Yes | Yes |
| 33510816 | 1 | NLLiVa | NL introd | 2020 | Adult | Yes | Yes |
| 36217869 | 1 | NLLiVa | NL introd | 2019 | Adult | Yes | Yes |
| 36293351 | 1 | NLLiVa | NL introd | 2019 | Adult | Yes | Yes |
| 36723083 | 1 | NLLiVa | NL introd | 2019 | Adult | Yes | Yes |
| 38884593 | 1 | NLLiVa | NL introd | 2019 | Adult | Yes | Yes |
| 38884606 | 1 | NLLiVa | NL introd | 2019 | Adult | Yes | Yes |
| 40217546 | 1 | NLLiVa | NL introd | 2020 | Adult | Yes | Yes |
| 40217951 | 1 | NLLiVa | NL introd | 2020 | Adult | Yes | Yes |
| 40220489 | 1 | NLLiVa | NL introd | 2020 | Adult | Yes | Yes |
| 40220518 | 1 | NLLiVa | NL introd | 2020 | Adult | Yes | Yes |
| 40224991 | 1 | NLLiVa | NL introd | 2020 | Adult | Yes | Yes |
| 40342179 | 1 | NLLiVa | NL introd | 2019 | Adult | Yes | Yes |
| 40389761 | 1 | NLLiVa | NL introd | 2020 | Adult | Yes | Yes |
| 40390349 | 1 | NLLiVa | NL introd | 2020 | Adult | Yes | Yes |
| 40390711 | 1 | NLLiVa | NL introd | 2020 | Adult | Yes | Yes |
| 72293201* | 1 | NLLiVa | NL introd | 2023 | Adult | No | No |
| 72293615 | 1 | NLLiVa | NL introd | 2023 | Adult | No | Yes |
| 38884681-1 | 1 | NLLiVa | NL introd | 2019 | Adult | Yes | Yes |
| 38884681-2 | 1 | NLLiVa | NL introd | 2019 | Adult | Yes | Yes |
| 39623512-1* | 1 | NLLiVa | NL introd | 2023 | Adult | No | No |
| 39623512-2* | 1 | NLLiVa | NL introd | 2023 | Adult | No | No |
| 72290182-1 | 1 | NLLiVa | NL introd | 2023 | Adult | No | Yes |
| 72290182-2 | 1 | NLLiVa | NL introd | 2023 | Adult | No | Yes |
| 72293009-1 | 1 | NLLiVa | NL introd | 2023 | Adult | No | Yes |
| 72293009-2 | 1 | NLLiVa | NL introd | 2023 | Adult | No | Yes |
| 72293561-1 | 1 | NLLiVa | NL introd | 2023 | Adult | No | Yes |
| 72293561-2* | 1 | NLLiVa | NL introd | 2023 | Adult | No | No |
| 32608627-1 | 2 | NLGeAa | NL introd | 2017 | Adult | Yes | Yes |
| 32608627-2 | 2 | NLGeAa | NL introd | 2017 | Adult | Yes | Yes |
| 32658431-1 | 2 | NLGeAa | NL introd | 2017 | Adult | Yes | Yes |
| 32906173-1 | 2 | NLGeAa | NL introd | 2017 | Adult | Yes | Yes |
| 32906210-1 | 2 | NLGeAa | NL introd | 2017 | Adult | Yes | Yes |
| 32906210-2* | 2 | NLGeAa | NL introd | 2017 | Adult | Yes | No |
| 32906210-3 | 2 | NLGeAa | NL introd | 2017 | Adult | Yes | Yes |
| 33208254-1 | 2 | NLGeAa | NL introd | 2017 | Adult | Yes | Yes |
| 33208270-1 | 2 | NLGeAa | NL introd | 2017 | Adult | Yes | Yes |
| 33208270-2 | 2 | NLGeAa | NL introd | 2017 | Adult | Yes | Yes |
| 33208270-3 | 2 | NLGeAa | NL introd | 2017 | Adult | Yes | Yes |
| 33238584-1 | 2 | NLGeAa | NL introd | 2017 | Adult | Yes | Yes |
| 33275860-1 | 2 | NLGeAa | NL introd | 2017 | Adult | Yes | Yes |
| 33785705-1 | 2 | NLGeAa | NL introd | 2017 | Adult | Yes | Yes |
| 33802480-1 | 2 | NLGeAa | NL introd | 2017 | Adult | Yes | Yes |
| 33803408-1 | 2 | NLGeAa | NL introd | 2017 | Adult | Yes | Yes |
| 33978339-1 | 2 | NLGeAa | NL introd | 2017 | Adult | Yes | Yes |
| 65223176-1* | 2 | NLGeAa | NL introd | 2023 | Adult | No | No |
| 65223176-2* | 2 | NLGeAa | NL introd | 2023 | Adult | No | No |
| 71890522-1* | 2 | NLGeAa | NL introd | 2023 | Adult | No | No |
| 71890522-2* | 2 | NLGeAa | NL introd | 2023 | Adult | No | No |
| 71890549-1* | 2 | NLGeAa | NL introd | 2023 | Adult | No | No |
| 71890549-2* | 2 | NLGeAa | NL introd | 2023 | Adult | No | No |
| 71890549-3* | 2 | NLGeAa | NL introd | 2023 | Adult | No | No |
| 71890549-4* | 2 | NLGeAa | NL introd | 2023 | Adult | No | No |
| 71890565-1 | 2 | NLGeAa | NL introd | 2023 | Adult | No | Yes |
| 71890565-2 | 2 | NLGeAa | NL introd | 2023 | Adult | No | Yes |
| 41367634 | 3 | NLLiSi | NL introd | 2020 | Adult | Yes | Yes |
| 41370200 | 3 | NLLiSi | NL introd | 2020 | Adult | Yes | Yes |
| 41370219 | 3 | NLLiSi | NL introd | 2020 | Adult | Yes | Yes |
| 32676101-1 | 3 | NLLiSi | NL introd | 2020 | Larva | Yes | Yes |
| 32676101-2 | 3 | NLLiSi | NL introd | 2020 | Larva | Yes | Yes |
| 33100884-1 | 3 | NLLiSi | NL introd | 2020 | Larva | Yes | Yes |
| 33100884-2 | 3 | NLLiSi | NL introd | 2020 | Larva | Yes | Yes |
| 33100884-3 | 3 | NLLiSi | NL introd | 2020 | Larva | Yes | Yes |
| 33100884-4 | 3 | NLLiSi | NL introd | 2020 | Larva | Yes | Yes |
| 33100884-5 | 3 | NLLiSi | NL introd | 2020 | Larva | Yes | Yes |
| 36543445-1 | 3 | NLLiSi | NL introd | 2020 | Larva | Yes | Yes |
| 36543445-2 | 3 | NLLiSi | NL introd | 2020 | Larva | Yes | Yes |
| 36572289-1 | 3 | NLLiSi | NL introd | 2020 | Larva | Yes | Yes |
| 36572289-2 | 3 | NLLiSi | NL introd | 2020 | Larva | Yes | Yes |
| 36572289-3 | 3 | NLLiSi | NL introd | 2020 | Larva | Yes | Yes |
| 36572289-4 | 3 | NLLiSi | NL introd | 2020 | Larva | Yes | Yes |
| 36573214-1 | 3 | NLLiSi | NL introd | 2020 | Larva | Yes | Yes |
| 36573214-2 | 3 | NLLiSi | NL introd | 2020 | Larva | Yes | Yes |
| 36573214-3 | 3 | NLLiSi | NL introd | 2020 | Larva | Yes | Yes |
| 36573214-4 | 3 | NLLiSi | NL introd | 2020 | Larva | Yes | Yes |
| 32772267 | 4 | NLZHAl | NL introd | 2019 | Adult | Yes | Yes |
| 36252023 | 4 | NLZHAl | NL introd | 2019 | Adult | Yes | Yes |
| 33262391-1 | 4 | NLZHAl | NL introd | 2019 | Larva | Yes | Yes |
| 33262391-2 | 4 | NLZHAl | NL introd | 2019 | Larva | Yes | Yes |
| 33262391-3 | 4 | NLZHAl | NL introd | 2019 | Larva | Yes | Yes |
| 33262391-4 | 4 | NLZHAl | NL introd | 2019 | Larva | Yes | Yes |
| 36726137-1 | 4 | NLZHAl | NL introd | 2019 | Larva | Yes | Yes |
| 36726137-2 | 4 | NLZHAl | NL introd | 2019 | Larva | Yes | Yes |
| 36726137-3 | 4 | NLZHAl | NL introd | 2019 | Larva | Yes | Yes |
| 36726137-4 | 4 | NLZHAl | NL introd | 2019 | Larva | Yes | Yes |
| 36952687-1 | 4 | NLZHAl | NL introd | 2019 | Larva | Yes | Yes |
| 36952687-2 | 4 | NLZHAl | NL introd | 2019 | Larva | Yes | Yes |
| 36952687-3 | 4 | NLZHAl | NL introd | 2019 | Larva | Yes | Yes |
| 36952687-4 | 4 | NLZHAl | NL introd | 2019 | Larva | Yes | Yes |
| 36954543-2 | 4 | NLZHAl | NL introd | 2019 | Larva | Yes | Yes |
| 36954543-3 | 4 | NLZHAl | NL introd | 2019 | Larva | Yes | Yes |
| 36954543-4 | 4 | NLZHAl | NL introd | 2019 | Larva | Yes | Yes |
| 36954551-1 | 4 | NLZHAl | NL introd | 2019 | Larva | Yes | Yes |
| 36954551-2 | 4 | NLZHAl | NL introd | 2019 | Larva | Yes | Yes |
| 38585649 | 5 | NLNHUi | NL introd | 2019 | Larva | Yes | Yes |
| 39616160 | 5 | NLNHUi | NL introd | 2019 | Adult | Yes | Yes |
| 39948325 | 5 | NLNHUi | NL introd | 2019 | Adult | Yes | Yes |
| 39975593 | 5 | NLNHUi | NL introd | 2019 | Adult | Yes | Yes |
| 40026408-1 | 5 | NLNHUi | NL introd | 2019 | Larva | Yes | Yes |
| 40026408-2 | 5 | NLNHUi | NL introd | 2019 | Larva | Yes | Yes |
| 40026408-3 | 5 | NLNHUi | NL introd | 2019 | Larva | Yes | Yes |
| 40026408-4 | 5 | NLNHUi | NL introd | 2019 | Larva | Yes | Yes |
| 40026408-5 | 5 | NLNHUi | NL introd | 2019 | Larva | Yes | Yes |
| 40026416-1 | 5 | NLNHUi | NL introd | 2019 | Larva | Yes | Yes |
| 40026416-2 | 5 | NLNHUi | NL introd | 2019 | Larva | Yes | Yes |
| 40026416-3 | 5 | NLNHUi | NL introd | 2019 | Larva | Yes | Yes |
| 40026416-4 | 5 | NLNHUi | NL introd | 2019 | Larva | Yes | Yes |
| 40026416-5 | 5 | NLNHUi | NL introd | 2019 | Larva | Yes | Yes |
| 40026416-6 | 5 | NLNHUi | NL introd | 2019 | Larva | Yes | Yes |
| 40026563-1 | 5 | NLNHUi | NL introd | 2019 | Larva | Yes | Yes |
| 40026563-2 | 5 | NLNHUi | NL introd | 2019 | Larva | Yes | Yes |
| 40026563-4 | 5 | NLNHUi | NL introd | 2019 | Larva | Yes | Yes |
| 40026563-5 | 5 | NLNHUi | NL introd | 2019 | Larva | Yes | Yes |
| 5484577-1 | 6 | NLGeVe | NL introd | 2016 | Adult | Yes | Yes |
| 5484577-2 | 6 | NLGeVe | NL introd | 2016 | Adult | Yes | Yes |
| 6123888-1 | 6 | NLGeVe | NL introd | 2016 | Adult | Yes | Yes |
| 6302624-1 | 6 | NLGeVe | NL introd | 2016 | Adult | Yes | Yes |
| 6400208-1 | 6 | NLGeVe | NL introd | 2016 | Adult | Yes | Yes |
| 6443689-1 | 6 | NLGeVe | NL introd | 2016 | Adult | Yes | Yes |
| 6499594-1 | 6 | NLGeVe | NL introd | 2016 | Adult | Yes | Yes |
| 6499658-1 | 6 | NLGeVe | NL introd | 2016 | Adult | Yes | Yes |
| 6499658-2 | 6 | NLGeVe | NL introd | 2016 | Adult | Yes | Yes |
| 6557774-1 | 6 | NLGeVe | NL introd | 2016 | Adult | Yes | Yes |
| 6762268-1 | 6 | NLGeVe | NL introd | 2016 | Adult | Yes | Yes |
| 6762268-2 | 6 | NLGeVe | NL introd | 2016 | Adult | Yes | Yes |
| 6762292-1 | 6 | NLGeVe | NL introd | 2016 | Adult | Yes | Yes |
| 6762305-1 | 6 | NLGeVe | NL introd | 2016 | Adult | Yes | Yes |
| 6768088-1 | 6 | NLGeVe | NL introd | 2016 | Adult | Yes | Yes |
| 6768088-2 | 6 | NLGeVe | NL introd | 2016 | Adult | Yes | Yes |
| 6768117-1 | 6 | NLGeVe | NL introd | 2016 | Adult | Yes | Yes |
| 6768133-1 | 6 | NLGeVe | NL introd | 2016 | Adult | Yes | Yes |
| 39789125 | 7 | NLDrAs | PoE tires | 2023 | Adult | No | Yes |
| 39789231 | 7 | NLDrAs | PoE tires | 2023 | Adult | No | Yes |
| 40038943* | 7 | NLDrAs | PoE tires | 2022 | Adult | No | No |
| 40220577 | 7 | NLDrAs | PoE tires | 2022 | Adult | No | Yes |
| 41209935 | 7 | NLDrAs | PoE tires | 2022 | Adult | No | Yes |
| 65514619 | 7 | NLDrAs | PoE tires | 2023 | Adult | No | Yes |
| 66097183 | 7 | NLDrAs | PoE tires | 2022 | Adult | No | Yes |
| 5616031-3 | 7 | NLDrAs | PoE tires | 2015 | Adult | No | Yes |
| 64818717-1 | 7 | NLDrAs | PoE tires | 2023 | Adult | No | Yes |
| 64818717-2 | 7 | NLDrAs | PoE tires | 2023 | Adult | No | Yes |
| 64819031-1 | 7 | NLDrAs | PoE tires | 2023 | Adult | No | Yes |
| 64819031-2 | 7 | NLDrAs | PoE tires | 2023 | Adult | No | Yes |
| 65946294-1 | 7 | NLDrAs | PoE tires | 2022 | Adult | No | Yes |
| 65946294-2 | 7 | NLDrAs | PoE tires | 2022 | Adult | No | Yes |
| 65946294-3 | 7 | NLDrAs | PoE tires | 2022 | Adult | No | Yes |
| 32671730-1 | 8 | NLLiWe | NL introd | 2017 | Adult | Yes | Yes |
| 33033925-1 | 8 | NLLiWe | NL introd | 2017 | Adult | Yes | Yes |
| 33628558-1 | 8 | NLLiWe | NL introd | 2018 | Adult | Yes | Yes |
| 33628566-1 | 8 | NLLiWe | NL introd | 2018 | Adult | Yes | Yes |
| 33632161-1 | 8 | NLLiWe | NL introd | 2017 | Adult | Yes | Yes |
| 33781173-1b | 8 | NLLiWe | NL introd | 2017 | Adult | Yes | Yes |
| 33781173-2b | 8 | NLLiWe | NL introd | 2017 | Adult | Yes | Yes |
| 33783152-1 | 8 | NLLiWe | NL introd | 2017 | Adult | Yes | Yes |
| 33970062-1 | 8 | NLLiWe | NL introd | 2017 | Adult | Yes | Yes |
| 34159650-1 | 8 | NLLiWe | NL introd | 2017 | Adult | Yes | Yes |
| 36374493-1 | 8 | NLLiWe | NL introd | 2018 | Adult | Yes | Yes |
| 6360779-1 | 8 | NLLiWe | NL introd | 2016 | Adult | Yes | Yes |
| 6556704-1 | 8 | NLLiWe | NL introd | 2016 | Adult | Yes | Yes |
| 41351966 | 9 | NLUtMo | PoE tires | 2022 | Adult | No | Yes |
| 42053928 | 9 | NLUtMo | PoE tires | 2023 | Adult | No | Yes |
| 42054314 | 9 | NLUtMo | PoE tires | 2023 | Adult | No | Yes |
| 42403289 | 9 | NLUtMo | PoE tires | 2022 | Adult | No | Yes |
| 65205410 | 9 | NLUtMo | PoE tires | 2023 | Adult | No | Yes |
| 65227291* | 9 | NLUtMo | PoE tires | 2023 | Adult | No | No |
| 65806400 | 9 | NLUtMo | PoE tires | 2023 | Adult | No | Yes |
| 66606697 | 9 | NLUtMo | PoE tires | 2023 | Adult | No | Yes |
| 41899630-1 | 9 | NLUtMo | PoE tires | 2022 | Adult | No | Yes |
| 41899630-2 | 9 | NLUtMo | PoE tires | 2022 | Adult | No | Yes |
| 65206384-1 | 9 | NLUtMo | PoE tires | 2023 | Adult | No | Yes |
| 65206384-2 | 9 | NLUtMo | PoE tires | 2023 | Adult | No | Yes |
| 6199428-1 | 10 | CHGuGu | Reference panel | 2016 | Adult | Yes | Yes |
| 6199428-10 | 10 | CHGuGu | Reference panel | 2016 | Adult | Yes | Yes |
| 6199428-4b | 10 | CHGuGu | Reference panel | 2016 | Adult | Yes | Yes |
| 6199428-5 | 10 | CHGuGu | Reference panel | 2016 | Adult | Yes | Yes |
| 6199428-6* | 10 | CHGuGu | Reference panel | 2016 | Adult | Yes | No |
| 6199428-7 | 10 | CHGuGu | Reference panel | 2016 | Adult | Yes | Yes |
| 6199428-8 | 10 | CHGuGu | Reference panel | 2016 | Adult | Yes | Yes |
| 6199428-9 | 10 | CHGuGu | Reference panel | 2016 | Adult | Yes | Yes |
| 6410932-3b* | 11 | ITERCo | Reference panel | 2016 | Adult | Yes | No |
| 6410932-4b | 11 | ITERCo | Reference panel | 2016 | Adult | Yes | Yes |
| 6410932-5 | 11 | ITERCo | Reference panel | 2016 | Adult | Yes | Yes |
| 6410940-1b | 11 | ITRaRa | Reference panel | 2016 | Adult | Yes | Yes |
| 6410940-2b | 11 | ITRaRa | Reference panel | 2016 | Adult | Yes | Yes |
| 6410940-3b* | 11 | ITRaRa | Reference panel | 2016 | Adult | Yes | No |
| 36489303-2 | 12 | NLNBBr | PoE Highway | 2020 | Egg | Yes | Yes |
| 36489303-3 | 12 | NLNBBr | PoE Highway | 2020 | Egg | Yes | Yes |
| 36489303-4 | 12 | NLNBBr | PoE Highway | 2020 | Egg | Yes | Yes |
| 36489303-5* | 12 | NLNBBr | PoE Highway | 2020 | Egg | Yes | No |
| 39775962-3 | 12 | NLNBBr | PoE Highway | 2020 | Egg | Yes | Yes |
| 5689812-2 | 13 | ESCaBa | Reference panel | 2016 | Adult | No | Yes |
| 5689812-3 | 13 | ESCaBa | Reference panel | 2016 | Adult | No | Yes |
| 5689820-4 | 13 | ESCaVa | Reference panel | 2016 | Adult | No | Yes |
| 5689820-5 | 13 | ESCaVa | Reference panel | 2016 | Adult | No | Yes |
| 5689820-6 | 13 | ESCaVa | Reference panel | 2016 | Adult | No | Yes |
| 34140298 | 14 | NLNHHa | PoE airport | 2018 | Adult | Yes | Yes |
| 36299067 | 14 | NLNHHa | PoE airport | 2019 | Adult | Yes | Yes |
| 36351461 | 14 | NLNHHa | PoE airport | 2018 | Adult | Yes | Yes |
| 38549058 | 14 | NLNHHa | PoE airport | 2018 | Adult | Yes | Yes |
| 33273793 | 15 | NLNBNe | NL introd | 2019 | Adult | Yes | Yes |
| 36882682 | 15 | NLNBNe | NL introd | 2019 | Adult | Yes | Yes |
| 39427791 | 15 | NLNBNe | NL introd | 2019 | Adult | Yes | Yes |
| 38879989-1 | 16 | NLDrHo | NL introd | 2019 | Adult | Yes | Yes |
| 39785909-1 | 16 | NLDrHo | NL introd | 2019 | Adult | Yes | Yes |
| 39785909-2 | 16 | NLDrHo | NL introd | 2019 | Adult | Yes | Yes |
| 5712701-2 | 17 | NLFlEm | PoE tires | 2014 | Adult | No | Yes |
| 5712875-2 | 17 | NLFlEm | PoE tires | 2014 | Adult | No | Yes |
| 6546450-1 | 17 | NLGeVe | PoE tires | 2016 | Adult | Yes | Yes |
| 32653681 | 18 | NLGeWe | NL introd | 2018 | Adult | Yes | Yes |
| 33631255 | 18 | NLGeWe | NL introd | 2018 | Adult | Yes | Yes |
| 36213851 | 18 | NLGeWe | NL introd | 2018 | Adult | Yes | Yes |
| 38764778 | 19 | NLZHNa | PoE auction | 2018 | Adult | Yes | Yes |
| 39583995 | 19 | NLZHNa | PoE auction | 2019 | Adult | Yes | Yes |
| 5962807-3 | 20 | NLZHLe | PoE Lucky bamboo | 2016 | Adult | No | Yes |
| 5962815-2 | 20 | NLZHLe | PoE Lucky bamboo | 2016 | Adult | No | Yes |
| 36256673 | 21 | NLNBEi | NL introd | 2018 | Adult | Yes | Yes |
| 5872617-2 | 22 | NLNHAm | PoE Lucky bamboo | 2015 | Adult | No | Yes |
| 6528068-3 | 23 | NLFlLe | PoE tires | 2016 | Adult | No | Yes |
| 32641777 | 24 | NLGeAr | NL introd | 2018 | Adult | Yes | Yes |

Supplementary Material 1: Supplementary Table S2. Microsatellite set for *Ae. albopictus* employed in the present study. For each microsatellite, the repeat unit, allele size range, forward (F) and reverse (R) primer sequences, primer concentration (C_0_), amplification multiplex panel (MPX) and primer label are shown. Locus Alb-tri-46 was discarded from analyses due to poor amplification. ^1^ *Ae. albopictus* microsatellites from Beebe et al. (2013); ^2^ *Ae. albopictus* microsatellites from Manni et al. (2015).

| Locus | Repeat unit | Allele size range (bp) | Primer sequences | C_0_ (μM) | MPX | Primer label (fluorescent dye) |
| --- | --- | --- | --- | --- | --- | --- |
| Aealbmic11^2^ | 3 | 188-230 | F: CTCTGCGTTCCGGTTCTATC  R: AGGCAACCTCTCGAATGAAA | 0.3 | 1 | 6-FAM |
| Aealbmic13^2^ | 3 | 132-171 | F: TCACACCATGGTCAAAGCAT  R: TGCTGAGTTGAATGGAAACG | 0.3 | 1 | HEX |
| Aealbmic3^2^ | 3 | 200-239 | F: ACCATACAGCCTGGAGTTCG  R: GGGGTTGTGTGAATTGTCGT | 0.3 | 1 | ATTO550 |
| Aealbmic7^2^ | 3 | 194-215 | F: ATAGACGGGAGTCGGTTCCT  R: TCCAACCGCTAGTGTCATCA | 0.3 | 1 | ATTO565 |
| Aealbmic9^2^ | 3 | 128-143 | F: GCGATGACAGTGGAACAAGA  R: GCTTGGCAGGGAACAAATTA | 0.5 | 1 | ATTO550 |
| Alb-tri-3^1^ | 3 | 123-153 | F: AGATGTGTCGCAATGCTTCC  R: GATTCGGTGATGTTGAGGCC | 0.3 | 1 | 6-FAM |
| Alb-tri-21^1^ | 3 | 137-206 | F: AGGGCTTCAATGGGTCTCTC  R: TGGTTATTAATACGGCGAGGC | 0.5 | 1 | ATTO565 |
| Aealbmic5^2^ | 3 | 136-214 | F: AACCCATCGAACACAGAAGG  R: GTACGGTTGACTCGCTGTGA | 0.3 | 2 | 6-FAM |
| Alb-tri-20^1^ | 3 | 165-201 | F: GTGCCGTTGATCATCCTGTC  R: TCCAGCACCGTGAGTAATCC | 0.3 | 2 | ATTO550 |
| Alb-tri-25^1^ | 3 | 257-278 | F: CCAACCAACAACCCAGGAAC  R: TACGATGCGCAACCATCATC | 0.3 | 2 | ATTO550 |
| Alb-tri-41^1^ | 3 | 134-155 | F: GATCGATTTGGGAGCTTCTG  R: GAACCTCTTCTCGCTTGGCT | 0.3 | 2 | ATTO565 |
| Alb-tri-46^1^ | 3 | 158-192 | F: TTCACAACATACGGAATCGC  R: GGTCCGGTGTAATAGCCTCC | 0.3 | 2 | HEX |
| Alb-tri-18^1^ | 3 | 250-280 | F: ACACAATTGCCGTTCAGCTC  R: CGTCTAATAGCTCCGGTCCC | 0.3 | 2 | HEX |
| Aealbmic4^2^ | 3 | 167-183 | F: ATCGCGGGTTTTCTATTCCT  R: ATCAACGAAACCGAAAGCAT | 0.3 | 3 | HEX |
| Aealbmic12^2^ | 3 | 155-182 | F: AGAGCCCTCGAAAAGAGAGC  R: AGCACTCATTCTTGGCTTGG | 0.3 | 3 | ATTO565 |
| Alb-tri-6^1^ | 3 | 164-219 | F: AGCACGAGTACAGAATGTGC  R: TGGCCTCCTACCGTTTATCTG | 0.3 | 3 | 6-FAM |
| Alb-tri-33^1^ | 3 | 137-182 | F: GGCTGCTGTTGTTGGTACG  R: CACGTTCAATCACCGGTTCC | 0.3 | 3 | HEX |
| Alb-tri-44^1^ | 3 | 173-212 | F: CACTCGCGCGTGTTCTTC  R: GACGCACCATCAGCATCATC | 0.3 | 3 | ATTO550 |
| Alb-tri-45^1^ | 3 | 120-150 | F: TTTCAGCTCGGTGTTATGGC  R: TGATGTTGATGATGATGACTACGA | 0.3 | 3 | ATTO550 |

**Supplementary Material 1: Supplementary Table S3.** Primer information used for the detection and identification of *Wolbachia* strains. Generic 81F and 691R primers were used for the detection of *Wolbachia* sp. in the *Ae. albopictus* samples. Primer combination 328F and 691R was used for the detection and identification of *Wolbachia* strain A (wAlbA) and primer combination 183F and 691R was used for the detection and identification of *Wolbachia* strain B (wAlbB).

| Primer name | Direction | Primer sequences (5’-3’) | Primer combinations | Product size |
| --- | --- | --- | --- | --- |
| 81F | Forward | TGG TCC AAT AAG TGA TGA AGA | 81F + 691R | wAlbA: 625 bp  wAlbB: 601 bp |
| 183F | Forward | AAG GAA CCG AAG TTC ATG | 183F + 691R | wAlbB: 500 bp |
| 328F | Forward | CCA GCA GAT ACT ATT GCG | 328F + 691R | wAlbA: 378 bp |
| 691R | Reverse | AAA AAT TAA ACG CTA CTC CA |  |  |

Supplementary Material 1: Supplementary S4. Final allelic dropout rate (‘DropRateEst’) and the rate of other kinds of genotyping errors (‘OtherErrorRateEst’) estimated using Colony.

| **Marker ID** | **StartDropRate** | **DropRateEst** | **DropRateCI95LB** | **DropRateCI95UB** | **StartOtherErrorRate** | **OtherErrorRateEst** | **OtherErrorRateCI95LB** | **OtherErrorRateCI95UB** |
| --- | --- | --- | --- | --- | --- | --- | --- | --- |
| Alb-tri-3 | 0.168 | 0.229 | 0.149 | 0.313 | 0.010 | 0.012 | 0.001 | 0.038 |
| Aealbmic11 | 0.197 | 0.243 | 0.171 | 0.318 | 0.010 | 0.014 | 0.001 | 0.052 |
| Aealbmic13 | 0.210 | 0.300 | 0.226 | 0.379 | 0.010 | 0.017 | 0.001 | 0.057 |
| Aealbmic9 | 0.154 | 0.153 | 0.089 | 0.211 | 0.010 | 0.000 | 0.000 | 0.024 |
| Aealbmic3 | 0.180 | 0.211 | 0.145 | 0.276 | 0.010 | 0.000 | 0.000 | 0.030 |
| Alb-tri-21 | 0.284 | 0.737 | 0.567 | 0.868 | 0.010 | 0.012 | 0.000 | 0.061 |
| Aealbmic7 | 0.091 | 0.087 | 0.044 | 0.141 | 0.010 | 0.022 | 0.003 | 0.058 |
| Aealbmic-5 | 0.174 | 0.221 | 0.153 | 0.294 | 0.010 | 0.007 | 0.000 | 0.041 |
| Alb-tri-18 | 0.175 | 0.251 | 0.171 | 0.338 | 0.010 | 0.016 | 0.000 | 0.052 |
| Alb-tri-20 | 0.095 | 0.092 | 0.051 | 0.137 | 0.010 | 0.000 | 0.000 | 0.030 |
| Alb-tri-25 | 0.043 | 0.020 | 0.000 | 0.065 | 0.010 | 0.000 | 0.000 | 0.023 |
| Alb-tri-41 | 0.164 | 0.278 | 0.187 | 0.381 | 0.010 | 0.037 | 0.004 | 0.086 |
| Alb-tri-6 | 0.114 | 0.093 | 0.046 | 0.146 | 0.010 | 0.024 | 0.007 | 0.051 |
| Alb-tri-33 | 0.051 | 0.050 | 0.020 | 0.085 | 0.010 | 0.000 | 0.000 | 0.021 |
| Aealbmic4 | 0.221 | 0.319 | 0.246 | 0.392 | 0.010 | 0.000 | 0.000 | 0.041 |
| Alb-tri-45 | 0.098 | 0.120 | 0.070 | 0.178 | 0.010 | 0.013 | 0.000 | 0.053 |
| Alb-tri-44 | 0.155 | 0.218 | 0.143 | 0.293 | 0.010 | 0.000 | 0.000 | 0.028 |
| Aealbmic12 | 0.152 | 0.168 | 0.113 | 0.204 | 0.010 | 0.000 | 0.000 | 0.015 |

**Supplementary Material 1: Supplementary Table S5.** Genetic diversity indices across *Ae. albopictus* locations, calculated based on 18 microsatellite markers. N-mean number of individuals per locus; Na-mean number of alleles per locus; Ne-mean number of effective alleles; I-Shannon’s information index; Ho-Observed heterozygosity; He-Expected heterozygosity; uHe-Unbiased expected heterozygosity; F-fixation index (inbreeding coefficient); %P-percentage of polymorphic loci. Summary of private alleles detected across populations. The majority of private alleles occurred at low frequencies (< 0.05), often confined to single loci.

| **Population** | **N** | **Na** | **Ne** | **I** | **Ho** | **He** | **uHe** | **F** | **%P** | **Private alleles/ Locus** | **Allele / Frequency** |
| --- | --- | --- | --- | --- | --- | --- | --- | --- | --- | --- | --- |
| NLLiVa | 23.22 | 4.72 | 2.79 | 1.12 | 0.39 | 0.58 | 0.59 | 0.34 | 100% | Alb-tri-3 | 122 / 0.04 |
|  |  |  |  |  |  |  |  |  |  | Aealbmic-5 | 205 / 0.02 |
| NLGeAa | 17.39 | 4.17 | 2.57 | 1.05 | 0.42 | 0.56 | 0.58 | 0.23 | 100% | - | - |
| NLLiSi | 19.33 | 4.33 | 2.85 | 1.15 | 0.46 | 0.61 | 0.63 | 0.28 | 100% | Aealbmic13 | 159 / 0.03 |
| NLZHAl | 18.78 | 4.33 | 2.99 | 1.17 | 0.48 | 0.62 | 0.64 | 0.25 | 100% | - | - |
| NLNHUi | 18.61 | 3.67 | 2.36 | 0.91 | 0.42 | 0.49 | 0.51 | 0.22 | 100% | Alb-tri-6 | 179 / 0.03 |
| NLGeVe | 17.39 | 4.61 | 3 | 1.19 | 0.53 | 0.63 | 0.65 | 0.18 | 100% | Alb-tri-6 | 164 / 0.03 |
|  |  |  |  |  |  |  |  |  |  |  | 188 / 0.03 |
|  |  |  |  |  |  |  |  |  |  |  | 197 / 0.03 |
| NLDrAs | 13.44 | 4.83 | 3.06 | 1.21 | 0.4 | 0.61 | 0.63 | 0.35 | 100% | Alb-tri-3 | 140 / 0.04 |
|  |  |  |  |  |  |  |  |  |  | Alb-tri-18 | 241 / 0.09 |
|  |  |  |  |  |  |  |  |  |  | Alb-tri-6 | 170 / 0.04 |
| NLLiWe | 12.72 | 5.11 | 3.34 | 1.29 | 0.46 | 0.65 | 0.68 | 0.3 | 100% | Alb-tri-45 | 116 / 0.04 |
| NLUtMo | 10.22 | 4.28 | 2.58 | 1.05 | 0.36 | 0.54 | 0.57 | 0.32 | 100% | - | - |
| CHGuGu | 6.22 | 3.33 | 2.42 | 0.9 | 0.36 | 0.49 | 0.53 | 0.22 | 88.90% | Alb-tri-18 | 229 / 0.14 |
| ITERCo + ITERRa | 3.72 | 2.78 | 2.22 | 0.83 | 0.33 | 0.49 | 0.57 | 0.35 | 88.90% | Alb-tri-18 | 250 / 0.25 |
| NLNBBr | 3.89 | 2.06 | 1.67 | 0.54 | 0.35 | 0.34 | 0.39 | 0.01 | 83.30% | - | - |
| ESCaBa + ESVaVa | 4.72 | 2.94 | 2.14 | 0.8 | 0.37 | 0.46 | 0.51 | 0.16 | 88.90% | - | - |
| NLNHHa^a^ | - | - | - | - | - | - | - | - | - | Alb-tri-6 | 146 / 0.13 |
| NLGeAr^a^ | - | - | - | - | - | - | - | - | - | Alb-tri-20 | 174 / 0.5 |
| NLNHAm^a^ | - | - | - | - | - | - | - | - | - | Aealbmic-5 | 175 / 0.5 |
| Total | 13.05 | 3.94 | 2.62 | 1.02 | 0.41 | 0.54 | 0.57 | 0.25 | 96.20% | - | - |

^a^ Genetic diversity parameters were not calculated for the populations with less than 5 specimens**.**

**Supplementary Material 1: Supplementary Table S6.** Error rates for the different kinship assignments, as estimated using CKMRsim. FPR: false positive rates; FNR: false negative rates; LogL ratio: Log-Likelihood ratio corresponding to the estimated kinship.

| **Kinship** | **FPR** | **FNR** | **LogL ratio** |
| --- | --- | --- | --- |
| PO vs U | 2,52 x10^-7^ | 0,774 | 12,5 |
| FS vs U | 2,61 x10^-7^ | 0,768 | 12,2 |
| HS vs U | 2,52 x10^-7^ | 0,995 | 9,17 |
| PO vs FS | 2,58 x10^-7^ | 1 | 6,98 |
| PO vs HS | 2,44 x10^-7^ | 1 | 6,76 |
| FS vs HS | 2,53 x10^-7^ | 0,994 | 9,36 |

**Supplementary Material 1: Supplementary Table S7.** Inferred 63 close-kin sample dyads and their *Wolbachia* strain infection status. Grey: close-kin dyads with concordant *Wolbachia* infection. Observations column: close-kin dyads showing multi-year kin at the same location, or close-kin between two locations.

| **Sample ID** | **Year** | **Location** | **Life stage** | ***Wolbachia*** | **Sample ID** | **Year** | **Location** | **Life stage** | ***Wolbachia*** | **Observations** |
| --- | --- | --- | --- | --- | --- | --- | --- | --- | --- | --- |
| 33275860-1 | 2017 | NL_Ge_Aa | adult | AB | 33785705-1 | 2017 | NL_Ge_Aa | adult | AB | - |
| 6123888-1 | 2016 | NL_Ge_Ve | adult | AB | 6499658-2 | 2016 | NL_Ge_Ve | adult | AB | - |
| 6762268-2 | 2016 | NL_Ge_Ve | adult | AB | 6762305-1 | 2016 | NL_Ge_Ve | adult | AB | - |
| 32653681 | 2018 | NL_Ge_We | adult | AB | 33631255 | 2018 | NL_Ge_We | adult | AB | - |
| 36573214-1 | 2020 | NL_Li_Si | larva | - | 36573214-4 | 2020 | NL_Li_Si | larva | - | - |
| 36573214-1 | 2020 | NL_Li_Si | larva | - | 36573214-2 | 2020 | NL_Li_Si | larva | - | - |
| 36572289-1 | 2020 | NL_Li_Si | larva | - | 36572289-4 | 2020 | NL_Li_Si | larva | - | - |
| 36572289-2 | 2020 | NL_Li_Si | larva | - | 36572289-4 | 2020 | NL_Li_Si | larva | - | - |
| 33100884-2 | 2020 | NL_Li_Si | larva | - | 33100884-3 | 2020 | NL_Li_Si | larva | AB | - |
| 32676101-1 | 2020 | NL_Li_Si | larva | - | 32676101-2 | 2020 | NL_Li_Si | larva | - | - |
| 36572289-1 | 2020 | NL_Li_Si | larva | - | 36572289-2 | 2020 | NL_Li_Si | larva | - | - |
| 36573214-1 | 2020 | NL_Li_Si | larva | - | 36573214-3 | 2020 | NL_Li_Si | larva | - | - |
| 40389761 | 2020 | NL_Li_Va | adult | AB | 40390711 | 2020 | NL_Li_Va | adult | AB | - |
| 40389761 | 2020 | NL_Li_Va | adult | AB | 40390349 | 2020 | NL_Li_Va | adult | - | - |
| 33510816 | 2020 | NL_Li_Va | adult | AB | 40390349 | 2020 | NL_Li_Va | adult | - | - |
| 32520617 | 2019 | NL_Li_Va | adult | B | 40389761 | 2020 | NL_Li_Va | adult | AB | multi-year kin at same residential site |
| 40342179 | 2019 | NL_Li_Va | adult | AB | 40390349 | 2020 | NL_Li_Va | adult | - | multi-year kin at same residential site |
| 33510816 | 2020 | NL_Li_Va | adult | AB | 40342179 | 2019 | NL_Li_Va | adult | AB | multi-year kin at same residential site |
| 32520617 | 2019 | NL_Li_Va | adult | B | 33510816 | 2020 | NL_Li_Va | adult | AB | multi-year kin at same residential site |
| 38884593 | 2019 | NL_Li_Va | adult | - | 38884681-2 | 2019 | NL_Li_Va | adult | AB | - |
| 33632161-1 | 2017 | NL_Li_We | adult | AB | 33783152-1 | 2017 | NL_Li_We | adult | AB | - |
| 6360779-1 | 2016 | NL_Li_We | adult | AB | 6556704-1 | 2016 | NL_Li_We | adult | AB | - |
| 33781173-1b | 2017 | NL_Li_We | adult | AB | 33783152-1 | 2017 | NL_Li_We | adult | AB | - |
| 33273793 | 2019 | NL_NB_Ne | adult | - | 39427791 | 2019 | NL_NB_Ne | adult | - | - |
| 36299067 | 2019 | NL_NH_Ha | adult | - | 36954543-2 | 2019 | NL_ZH_Al | larva | - | Same year kin PoE ↔ residential |
| 36299067 | 2019 | NL_NH_Ha | adult | - | 36952687-3 | 2019 | NL_ZH_Al | larva | AB | Same year kin PoE ↔ residential |
| 40026408-3 | 2019 | NL_NH_Ui | larva | B | 40026408-4 | 2019 | NL_NH_Ui | larva | - | - |
| 40026416-6 | 2019 | NL_NH_Ui | larva | - | 40026536-1 | 2019 | NL_NH_Ui | larva | - | - |
| 39616160 | 2019 | NL_NH_Ui | adult | AB | 40026416-4 | 2019 | NL_NH_Ui | larva | A | - |
| 39616160 | 2019 | NL_NH_Ui | adult | AB | 40026408-4 | 2019 | NL_NH_Ui | larva | - | - |
| 40026408-4 | 2019 | NL_NH_Ui | larva | - | 40026536-1 | 2019 | NL_NH_Ui | larva | - | - |
| 40026408-3 | 2019 | NL_NH_Ui | larva | B | 40026536-1 | 2019 | NL_NH_Ui | larva | - | - |
| 40026416-2 | 2019 | NL_NH_Ui | larva | - | 40026536-2 | 2019 | NL_NH_Ui | larva | - | - |
| 40026408-2 | 2019 | NL_NH_Ui | larva | B | 40026408-4 | 2019 | NL_NH_Ui | larva | - | - |
| 40026408-2 | 2019 | NL_NH_Ui | larva | B | 40026408-3 | 2019 | NL_NH_Ui | larva | B | - |
| 39616160 | 2019 | NL_NH_Ui | adult | AB | 40026536-1 | 2019 | NL_NH_Ui | larva | - | - |
| 40026416-4 | 2019 | NL_NH_Ui | larva | A | 40026416-5 | 2019 | NL_NH_Ui | larva | AB | - |
| 39616160 | 2019 | NL_NH_Ui | adult | AB | 40026408-3 | 2019 | NL_NH_Ui | larva | B | - |
| 40026416-1 | 2019 | NL_NH_Ui | larva | - | 40026536-1 | 2019 | NL_NH_Ui | larva | - | - |
| 39616160 | 2019 | NL_NH_Ui | adult | AB | 40026416-6 | 2019 | NL_NH_Ui | larva | - | - |
| 40026408-4 | 2019 | NL_NH_Ui | larva | - | 40026536-4 | 2019 | NL_NH_Ui | larva | AB | - |
| 40026408-4 | 2019 | NL_NH_Ui | larva | - | 40026416-1 | 2019 | NL_NH_Ui | larva | - | - |
| 40026416-4 | 2019 | NL_NH_Ui | larva | A | 40026416-6 | 2019 | NL_NH_Ui | larva | - | - |
| 40026416-6 | 2019 | NL_NH_Ui | larva | - | 40026536-2 | 2019 | NL_NH_Ui | larva | - | - |
| 40026408-3 | 2019 | NL_NH_Ui | larva | B | 40026536-4 | 2019 | NL_NH_Ui | larva | AB | - |
| 40026408-3 | 2019 | NL_NH_Ui | larva | B | 40026416-1 | 2019 | NL_NH_Ui | larva | - | - |
| 40026416-4 | 2019 | NL_NH_Ui | larva | A | 40026536-2 | 2019 | NL_NH_Ui | larva | - | - |
| 41899630-2 | 2022 | NL_Ut_Mo | adult | - | 42054314 | 2023 | NL_Ut_Mo | adult | - | multi-year kin at same PoE site |
| 36726137-1 | 2019 | NL_ZH_Al | larva | - | 36726137-2 | 2019 | NL_ZH_Al | larva | - | - |
| 36954543-4 | 2019 | NL_ZH_Al | larva | AB | 36954551-1 | 2019 | NL_ZH_Al | larva | AB | - |
| 36726137-3 | 2019 | NL_ZH_Al | larva | - | 36726137-4 | 2019 | NL_ZH_Al | larva | - | - |
| 36954543-3 | 2019 | NL_ZH_Al | larva | AB | 36954543-4 | 2019 | NL_ZH_Al | larva | AB | - |
| 33262391-3 | 2019 | NL_ZH_Al | larva | AB | 36726137-4 | 2019 | NL_ZH_Al | larva | - | - |
| 33262391-4 | 2019 | NL_ZH_Al | larva | AB | 36726137-1 | 2019 | NL_ZH_Al | larva | - | - |
| 36726137-1 | 2019 | NL_ZH_Al | larva | - | 36726137-4 | 2019 | NL_ZH_Al | larva | - | - |
| 33262391-2 | 2019 | NL_ZH_Al | larva | - | 33262391-3 | 2019 | NL_ZH_Al | larva | AB | - |
| 33262391-3 | 2019 | NL_ZH_Al | larva | AB | 36726137-3 | 2019 | NL_ZH_Al | larva | - | - |
| 33262391-4 | 2019 | NL_ZH_Al | larva | AB | 36726137-3 | 2019 | NL_ZH_Al | larva | - | - |
| 33262391-4 | 2019 | NL_ZH_Al | larva | AB | 36726137-4 | 2019 | NL_ZH_Al | larva | - | - |
| 36726137-2 | 2019 | NL_ZH_Al | larva | - | 36726137-4 | 2019 | NL_ZH_Al | larva | - | - |
| 36726137-1 | 2019 | NL_ZH_Al | larva | - | 36726137-3 | 2019 | NL_ZH_Al | larva | - | - |
| 36726137-2 | 2019 | NL_ZH_Al | larva | - | 36726137-3 | 2019 | NL_ZH_Al | larva | - | - |
| 33262391-2 | 2019 | NL_ZH_Al | larva | - | 36726137-3 | 2019 | NL_ZH_Al | larva | - | - |

Supplementary Material 1: Supplementary Table S8. Summary of genetic diversity across 18 microsatellite loci in 13 *Ae. albopictus* sampling locations that met the minimum sample size of five individuals. Measures include total genetic diversity (Ht), mean expected heterozygosity (He), mean observed heterozygosity (Ho), inbreeding coefficients within subpopulations (Fis), total inbreeding coefficient (Fit), genetic differentiation among populations (Fst), and estimated gene flow (Nm). Locus Alb-tri-46 was discarded from analyses due to poor amplification.

| **Locus** | **Ht** | **Mean He** | **Mean Ho** | **Fis** | **Fit** | **Fst** | **Nm** |
| --- | --- | --- | --- | --- | --- | --- | --- |
| **Alb-tri-3** | 0.52 | 0.46 | 0.29 | 0.36 | 0.44 | 0.12 | 1.88 |
| **Aealbmic11** | 0.77 | 0.65 | 0.44 | 0.32 | 0.44 | 0.16 | 1.27 |
| **Aealbmic13** | 0.71 | 0.61 | 0.42 | 0.32 | 0.41 | 0.14 | 1.51 |
| **Aealbmic9** | 0.72 | 0.62 | 0.46 | 0.27 | 0.36 | 0.13 | 1.71 |
| **Aealbmic3** | 0.74 | 0.52 | 0.32 | 0.38 | 0.56 | 0.30 | 0.59 |
| **Alb-tri-21** | 0.34 | 0.19 | 0.05 | 0.77 | 0.87 | 0.42 | 0.35 |
| **Aealbmic7** | 0.68 | 0.59 | 0.55 | 0.06 | 0.19 | 0.14 | 1.55 |
| **Aealbmic-5** | 0.70 | 0.60 | 0.39 | 0.35 | 0.45 | 0.15 | 1.39 |
| **Alb-tri-18** | 0.63 | 0.50 | 0.28 | 0.44 | 0.55 | 0.20 | 0.98 |
| **Alb-tri-20** | 0.76 | 0.66 | 0.59 | 0.11 | 0.22 | 0.13 | 1.75 |
| **Alb-tri-25** | 0.61 | 0.50 | 0.48 | 0.03 | 0.21 | 0.19 | 1.09 |
| **Alb-tri-41** | 0.47 | 0.43 | 0.27 | 0.38 | 0.43 | 0.09 | 2.56 |
| **Alb-tri-6** | 0.79 | 0.64 | 0.56 | 0.12 | 0.29 | 0.19 | 1.09 |
| **Alb-tri-33** | 0.58 | 0.51 | 0.59 | -0.16 | -0.02 | 0.12 | 1.80 |
| **Aealbmic4** | 0.80 | 0.65 | 0.34 | 0.49 | 0.58 | 0.19 | 1.10 |
| **Alb-tri-45** | 0.73 | 0.60 | 0.52 | 0.13 | 0.29 | 0.18 | 1.15 |
| **Alb-tri-44** | 0.50 | 0.40 | 0.25 | 0.36 | 0.49 | 0.21 | 0.93 |
| **Aealbmic12** | 0.81 | 0.68 | 0.59 | 0.13 | 0.27 | 0.16 | 1.33 |

**Supplementary Material 1: Supplementary Table S9.** Pairwise Nei’s Genetic Distance and Genetic Identity among 13 *Ae. albopictus* sampling locations that met the minimum sample size of five individuals from the Netherlands (10), one location from China (1), one from Italy (IT = ITERCo and ITERRa), and one from Spain (ES = ESCaBa and ESVaVa). The lower diagonal entries represent Nei’s genetic distance (D), the upper diagonal entries represent Nei’s genetic identity (I).

| **Location** | **NLLiVa** | **NLGeAa** | **NLLiSi** | **NLZHAl** | **NLNHUi** | **NLGeVe** | **NLDrAs** | **NLLiWe** | **NLUtMo** | **CHGuGu** | **IT** | **NLNBBr** | **ES** |
| --- | --- | --- | --- | --- | --- | --- | --- | --- | --- | --- | --- | --- | --- |
| **NLLiVa** |  | 0.77 | 0.78 | 0.78 | 0.64 | 0.74 | 0.82 | 0.83 | 0.80 | 0.66 | 0.56 | 0.76 | 0.81 |
| **NLGeAa** | 0.27 |  | 0.80 | 0.78 | 0.75 | 0.82 | 0.90 | 0.81 | 0.86 | 0.76 | 0.72 | 0.78 | 0.81 |
| **NLLiSi** | 0.25 | 0.23 |  | 0.74 | 0.66 | 0.72 | 0.86 | 0.81 | 0.86 | 0.74 | 0.66 | 0.78 | 0.81 |
| **NLZHAl** | 0.25 | 0.24 | 0.29 |  | 0.62 | 0.71 | 0.78 | 0.76 | 0.72 | 0.66 | 0.57 | 0.66 | 0.64 |
| **NLNHUi** | 0.44 | 0.29 | 0.42 | 0.48 |  | 0.66 | 0.69 | 0.62 | 0.66 | 0.55 | 0.67 | 0.66 | 0.66 |
| **NLGeVe** | 0.31 | 0.20 | 0.33 | 0.35 | 0.42 |  | 0.80 | 0.79 | 0.76 | 0.72 | 0.71 | 0.76 | 0.70 |
| **NLDrAs** | 0.19 | 0.10 | 0.15 | 0.24 | 0.37 | 0.22 |  | 0.87 | 0.92 | 0.81 | 0.75 | 0.82 | 0.85 |
| **NLLiWe** | 0.19 | 0.21 | 0.21 | 0.28 | 0.47 | 0.24 | 0.14 |  | 0.83 | 0.76 | 0.67 | 0.78 | 0.76 |
| **NLUtMo** | 0.22 | 0.16 | 0.15 | 0.33 | 0.42 | 0.28 | 0.08 | 0.18 |  | 0.82 | 0.71 | 0.84 | 0.86 |
| **CHGuGu** | 0.41 | 0.28 | 0.31 | 0.42 | 0.60 | 0.33 | 0.22 | 0.28 | 0.20 |  | 0.69 | 0.71 | 0.72 |
| **IT** | 0.58 | 0.33 | 0.42 | 0.56 | 0.40 | 0.34 | 0.29 | 0.39 | 0.34 | 0.37 |  | 0.67 | 0.66 |
| **NLNBBr** | 0.27 | 0.24 | 0.24 | 0.41 | 0.42 | 0.27 | 0.20 | 0.25 | 0.17 | 0.34 | 0.41 |  | 0.74 |
| **ES** | 0.21 | 0.22 | 0.21 | 0.45 | 0.42 | 0.35 | 0.17 | 0.27 | 0.16 | 0.32 | 0.42 | 0.30 |  |

**Supplementary Material 1: Supplementary Fig. S1** Principal Coordinates Analysis (PCoA) based on Codominant Genotypic Distance for 200 *Ae. albopictus* individuals from 26 locations. The dataset includes one location from China (CHGuGu), two from Spain (ESCaBa, ESVaVa), and two from Italy (ITERCo, ITERRa), with the remaining locations sampled across the Netherlands. The first two axes explain 34.09% of the total genetic variation (PCoA1: 20.41%, PCoA2: 13.68%). Clustering reflects genetic similarities and differences among sampling locations.


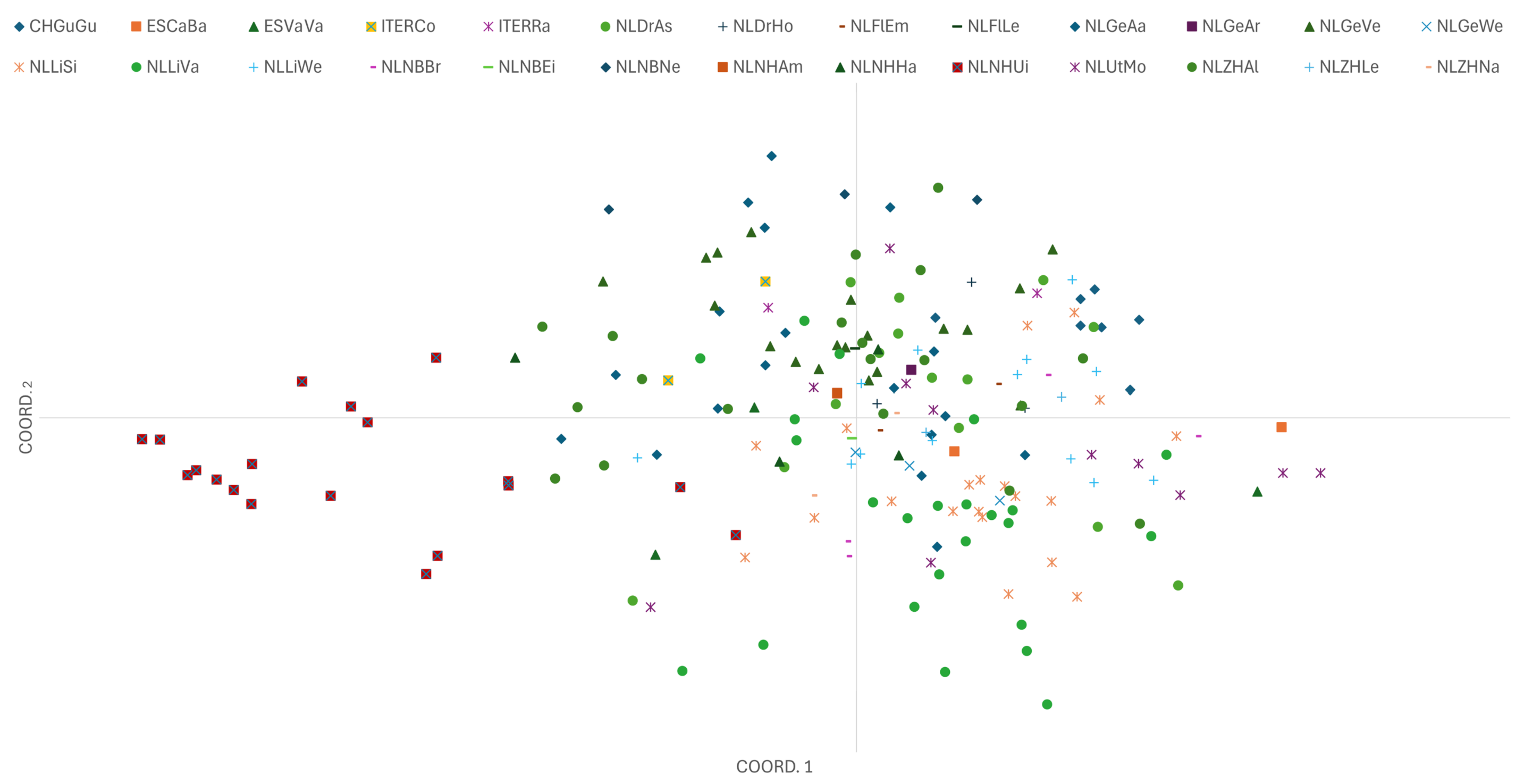


**Supplementary Material 1: Supplementary Fig. S2.** GenePlot visualization for localities with more than five individuals resampled over

| 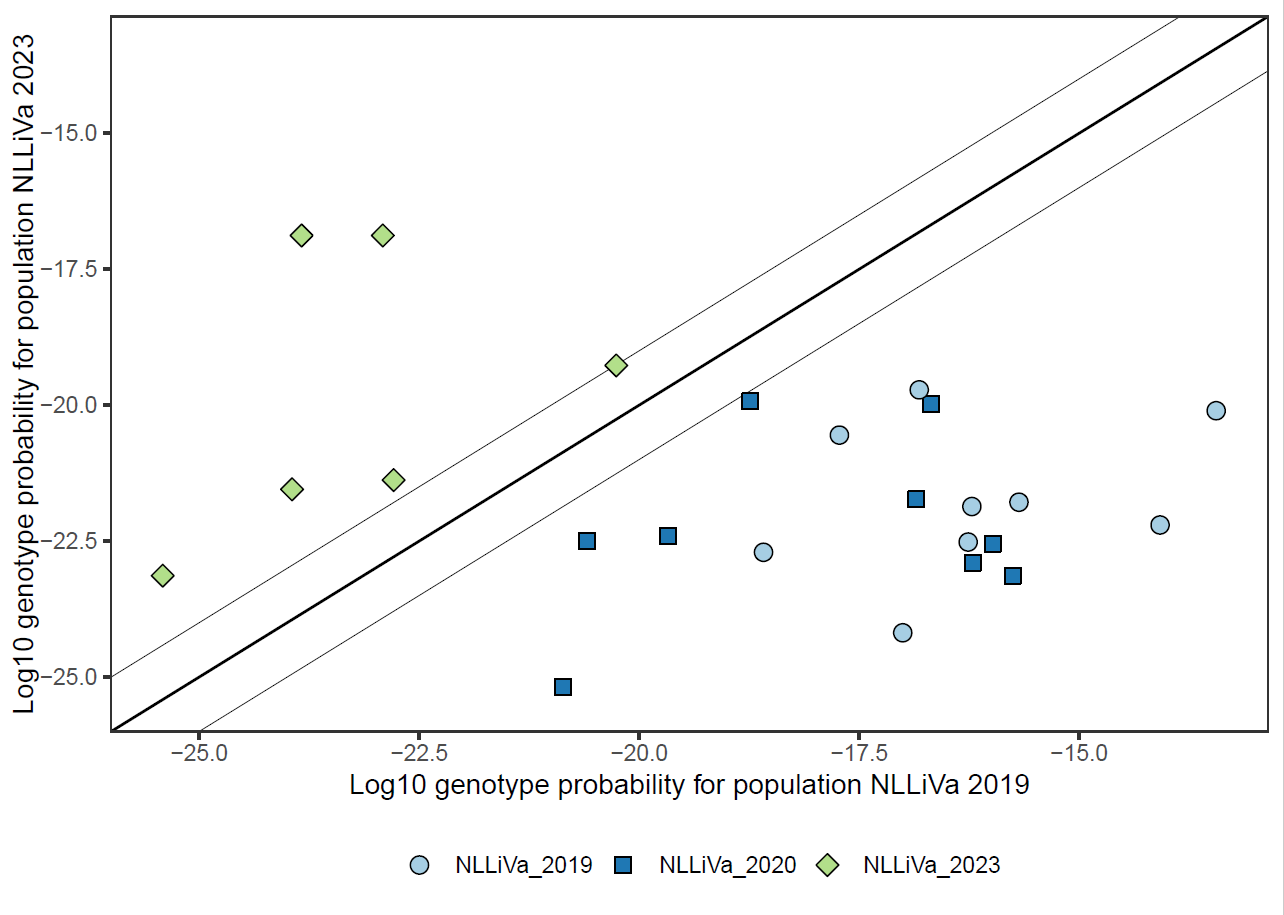 | 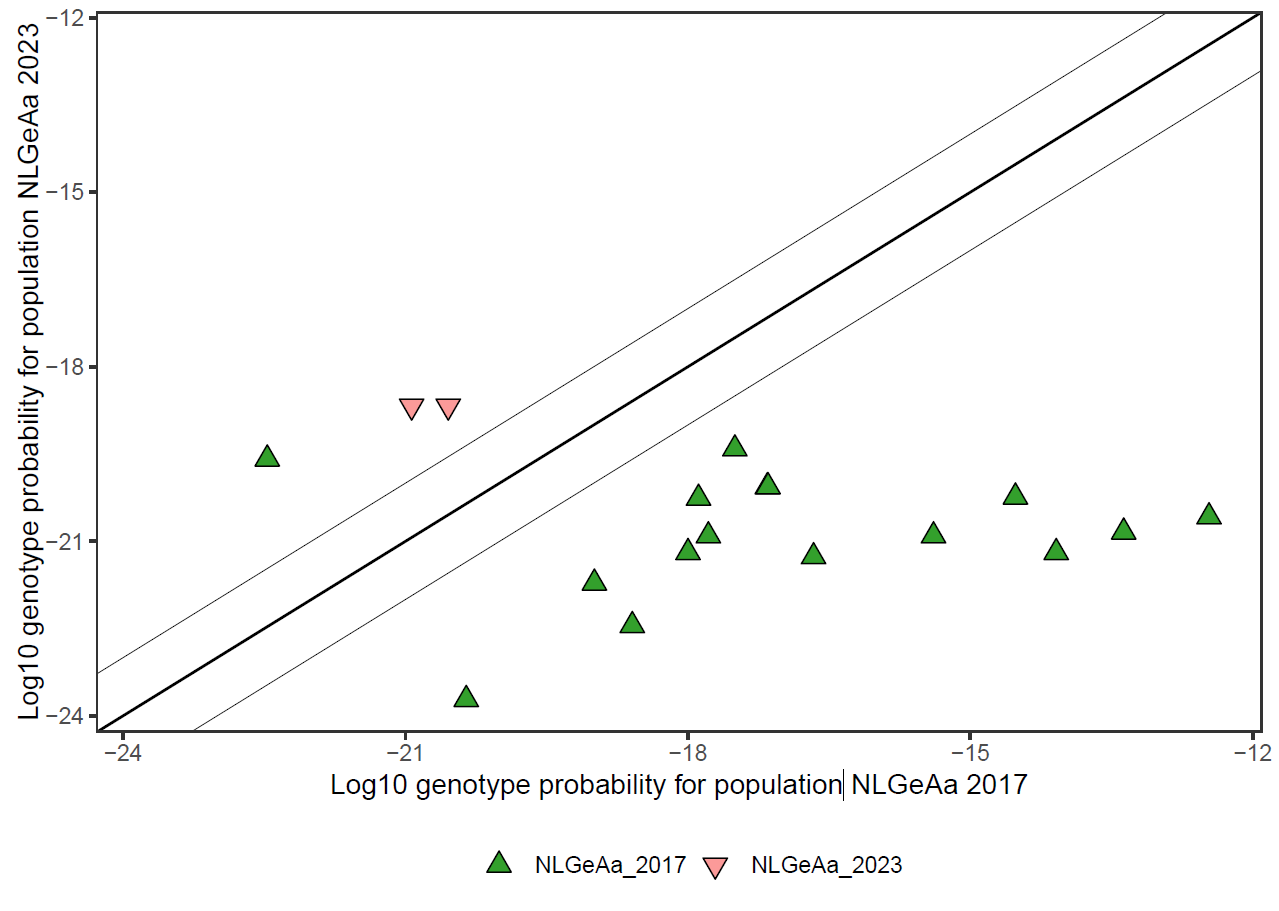 | 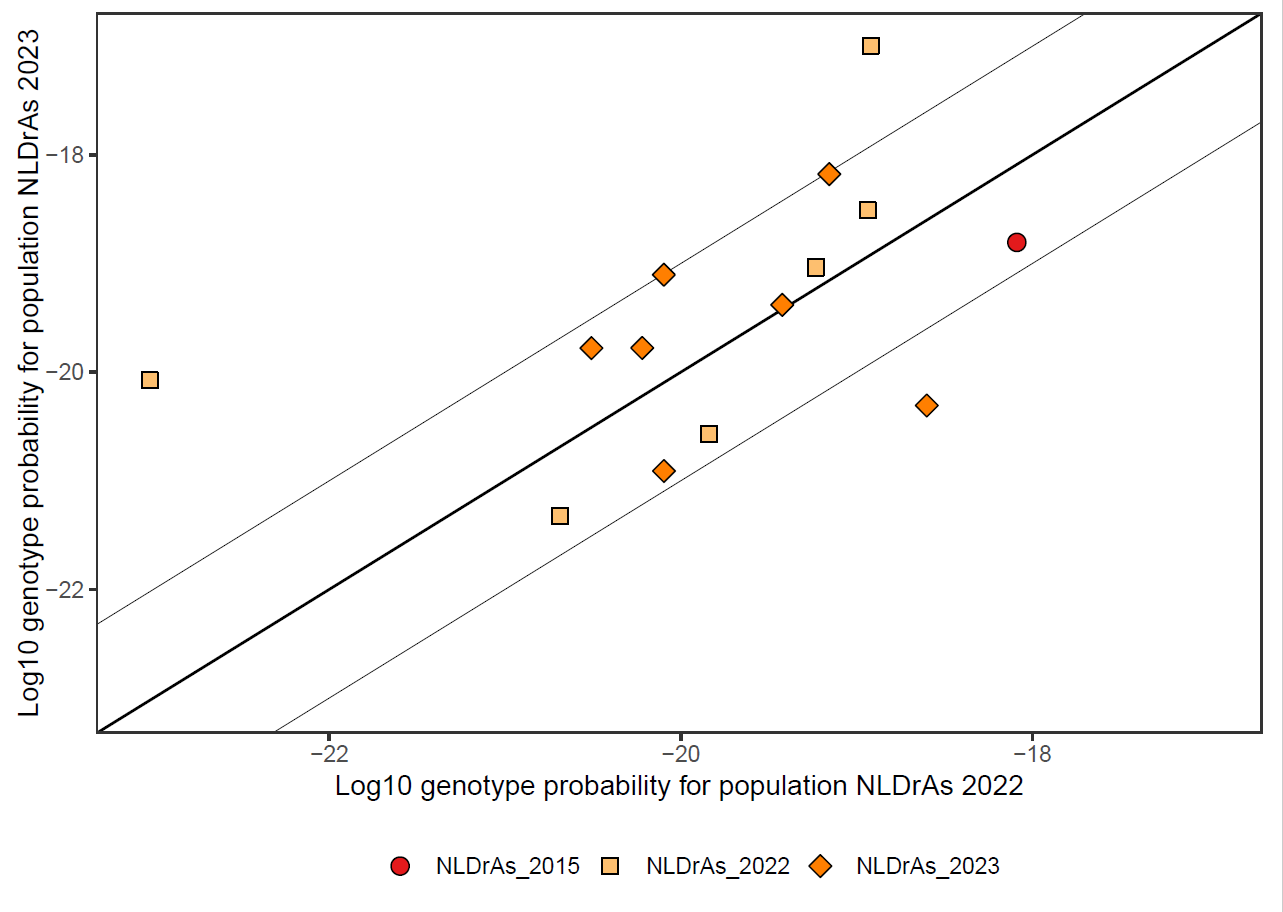 |
| --- | --- | --- |
| GenePlot for NLLiVa in 2019 versus 2023, assigning 2020 as free agent | GenePlot for NLGeAa in 2017 versus 2023 | GenePlot for NLDrAs in 2022 versus 2023, assigning 2015 as free agent |
| 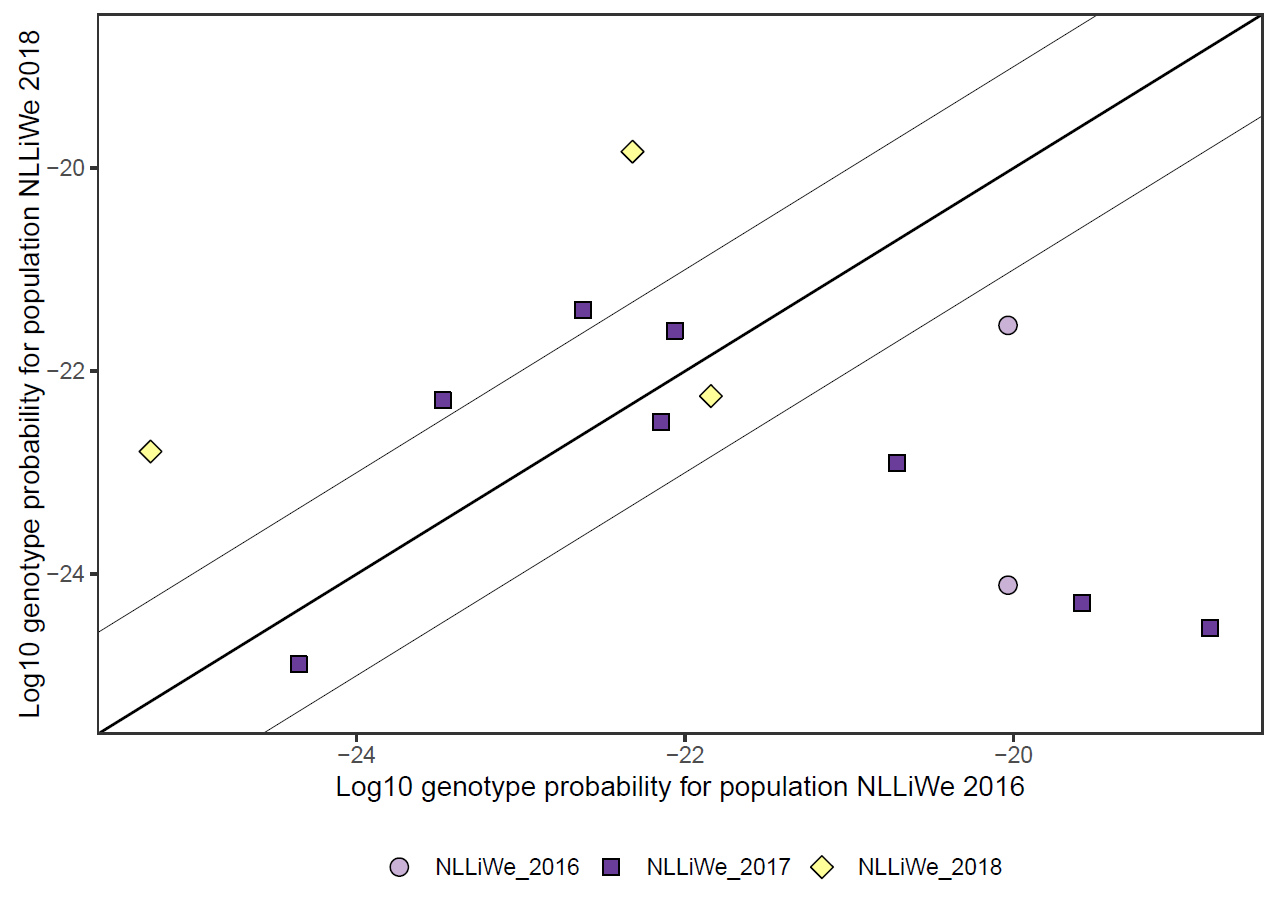 | 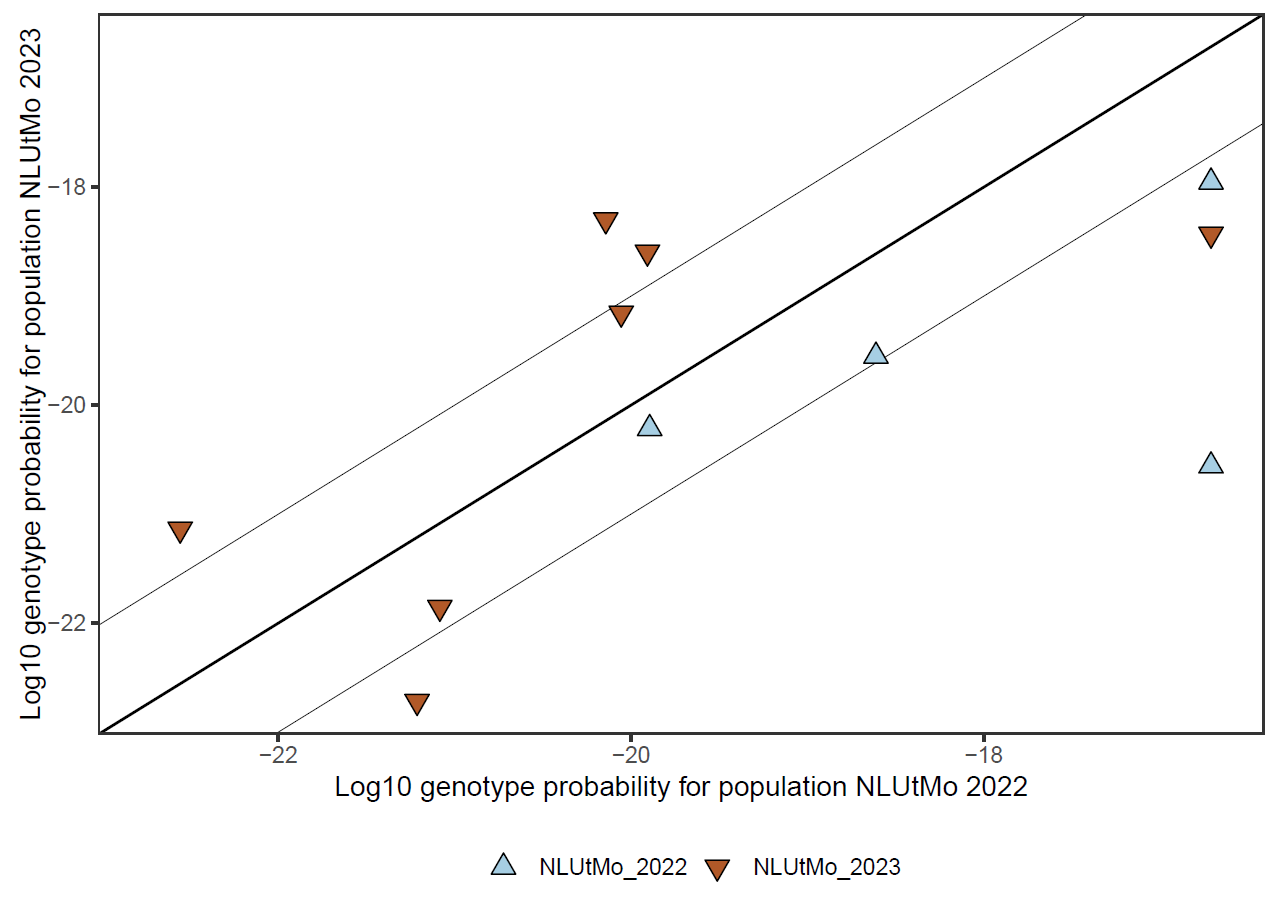 |  |
| GenePlot for NLLiWe in 2016 versus 2018, assigning 2017 as free agent | GenePlot for NLUtMo in 2022 versus 2023 |  |

multiple years
